# Discovery of a novel iota carrageenan sulfatase isolated from the marine bacterium *Pseudoalteromonas carrageenovora*

**DOI:** 10.3389/fchem.2014.00067

**Published:** 2014-08-26

**Authors:** Sabine M. Genicot, Agnès Groisillier, Hélène Rogniaux, Laurence Meslet-Cladière, Tristan Barbeyron, William Helbert

**Affiliations:** ^1^UMR 8227, Integrative Biology of Marine Models, Station Biologique de Roscoff, Sorbonne Universités, UPMC Université Paris 06Roscoff, France; ^2^UMR 8227, Centre National de la Recherche Scientifique, Integrative Biology of Marine Models, Station Biologique de RoscoffRoscoff, France; ^3^INRA, UR1268 Biopolymers Interactions AssembliesNantes, France; ^4^EA3882, LUBEM, Technopôle Brest-Iroise, Université de Bretagne OccidentalePlouzané, France; ^5^Centre de Recherches sur les Macromolécules Végétales (CERMAV, UPR-CNRS 5301), Université Joseph Fourier, and Member of the Institut de Chimie Moléculaire de Grenoble (ICMG, FR-CNRS 2607)Grenoble, France

**Keywords:** sulfatase, novel family, iota carrageenan, marine bacteria, sulfated polysaccharides, bioconversion

## Abstract

Carrageenans are sulfated polysaccharides extracted from the cell wall of some marine red algae. These polysaccharides are widely used as gelling, stabilizing, and viscosifying agents in the food and pharmaceutical industries. Since the rheological properties of these polysaccharides depend on their sulfate content, we screened several isolated marine bacteria for carrageenan specific sulfatase activity, in the aim of developing enzymatic bioconversion of carrageenans. As a result of the screening, an iota-carrageenan sulfatase was detected in the cell-free lysate of the marine bacterium *Pseudoalteromonas carrageenovora* strain Psc^T^. It was purified through Phenyl Sepharose and Diethylaminoethyl Sepharose chromatography. The pure enzyme, *Psc* ι-CgsA, was characterized. It had a molecular weight of 115.9 kDaltons and exhibited an optimal activity/stability at pH ~8.3 and at 40 ± 5°C. It was inactivated by phenylmethylsulfonyl fluoride but not by ethylene diamine tetraacetic acid. *Psc* ι-CgsA specifically catalyzes the hydrolysis of the 4-S sulfate of iota-carrageenan. The purified enzyme could transform iota-carrageenan into hybrid iota-/alpha- or pure alpha-carrageenan under controlled conditions. The gene encoding *Psc* ι-CgsA, a protein of 1038 amino acids, was cloned into *Escherichia coli*, and the sequence analysis revealed that *Psc* ι-CgsA has more than 90% sequence identity with a putative uncharacterized protein Q3IKL4 from the marine strain *Pseudoalteromonas haloplanktis* TAC 125, but besides this did not share any homology to characterized sulfatases. Phylogenetic studies show that *P. carrageenovora* sulfatase thus represents the first characterized member of a new sulfatase family, with a C-terminal domain having strong similarity with the superfamily of amidohydrolases, highlighting the still unexplored diversity of marine polysaccharide modifying enzymes.

## Introduction

Although sulfated biomolecules are present throughout the tree of life in terrestrial and marine environments, most of the sulfated carbohydrates are found in the marine environment, as illustrated by the structural diversity of sulfated polysaccharides encountered in the cell wall of macroalgae (Lahaye and Robic, 2007; Pomin and Mourao, 2008; Popper et al., 2011; Usov, 2011). As such polysaccharides are absent in the cell wall of fresh water and land plants; their loss has been postulated to be a consequence of the land colonization (Michel et al., 2010). These polysaccharides are thought to be involved in the phenomena of ionic and osmotic regulations. They are supposed to confer to the algae resistance to water currents and, due to their highly soluble nature, they allow water retention thereby slowing the drying of the algae at low tide (Kloareg and Quatrano, 1988).

**Figure 1:**
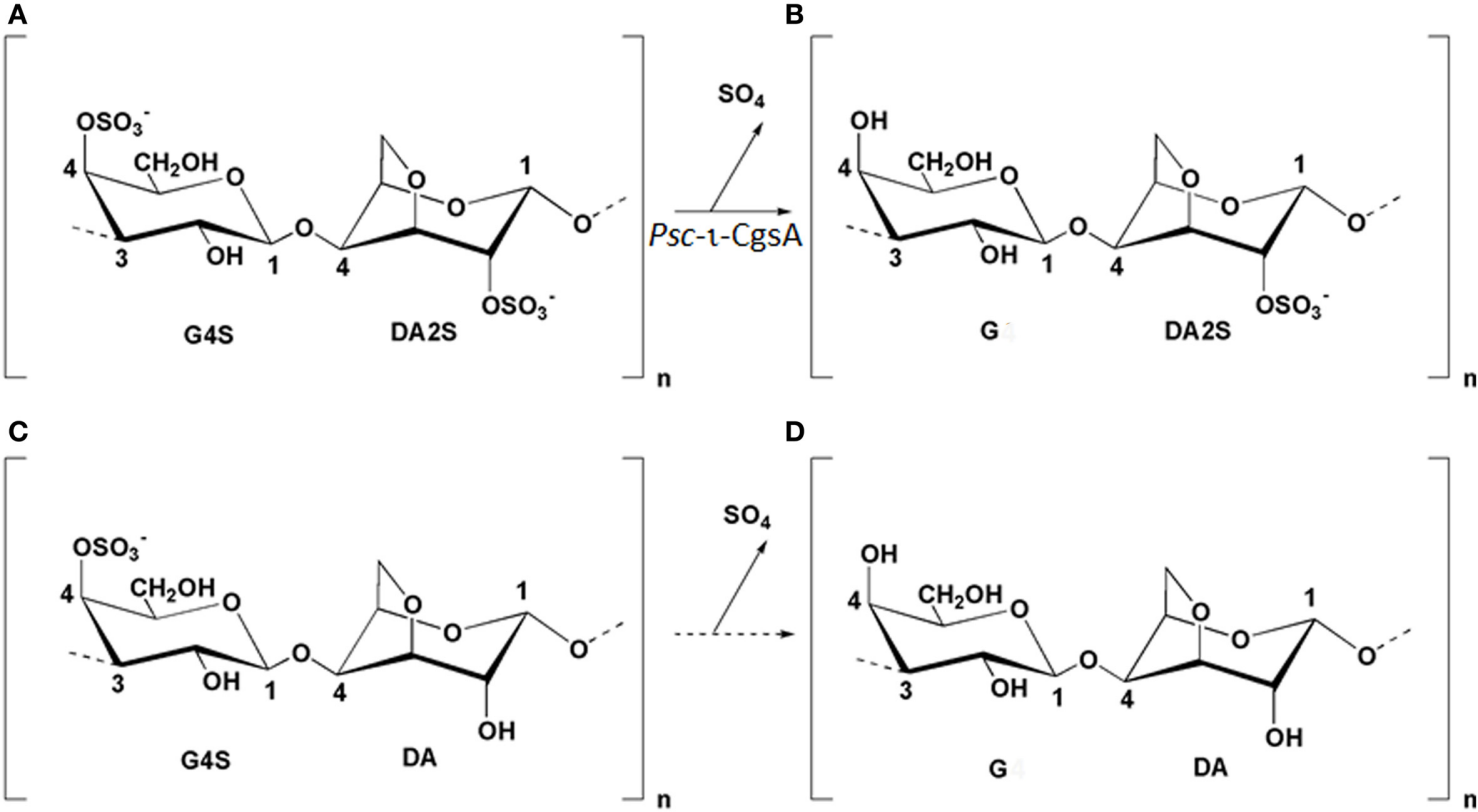
**Structure of the idealized repeating units of ι-(G4S-DA2S) (A), α-(G-DA2S) (B), κ-(G4S-DA) (C), and β-(G-DA) (D) carrageenans**. The arrow between G4S-DA2S and G-DA2S illustrates the reaction catalyzed by *Psc* ι-CgsA. Enzymatic production of β-carrageenan **(D)** from κ-carrageenan **(C)** has not been yet demonstrated and is represented by a dashed arrow.

The cell wall sulfated galactans of the red algae, referred to as agars or carrageenans, consist of a linear backbone of galactose residues linked by alternating β-1,4 and α-1,3 glycosidic bonds. While all the β-linked residues are in the D configuration (G monomer), the α-linked galactose units are in the L configuration in agars (L monomer) and in the D configuration in carrageenans (D monomer). In carrageenans, the repeating disaccharide units are classified according to the number and the position of ester sulfate (S) and by the presence of a 3,6-anhydro-bridge (DA) in the 4-linked residue (Knutsen et al., 1994). DA units are found in gelling carrageenans such as the kappa (κ; G4S-DA) and the iota (ι; G4S-DA2S)-carrageenan (Figure 1). Other substitutions, such as methyl or pyruvate groups, have also been observed, increasing the diversity of carrageenans which depends also on the algal source, the growth conditions and the extraction procedures (Pereira and Mesquita, 2004; Pereira et al., 2009). The physico-chemical properties of carrageenans, which are extensively used as thickeners and stabilizers in the food and cosmetic industries (de Ruiter and Rudolph, 1997), depend on their molecular weight, the occurrence of anhydrogalactose and their sulfate content and it is well established that higher levels of ester sulfate induce a decrease of the gel strength (Necas and Bartosikova, 2013). The use of specific enzymes to modify the sulfate pattern of carrageenans would therefore offer a biotechnological approach to control their sulfate content and thereby their rheological properties.

The heterogeneous structure of carrageenans, their gelling properties, and their interactions with the other components of red algal cell walls challenge the microorganisms using these polymers as carbon and energy sources. To breakdown the complex polysaccharides, marine bacteria secrete specific glycoside hydrolases (GHs), referred to as agarases and carrageenases, which catalyze the hydrolysis of the β-1,4 glycosidic bond between two galactopyranose units of their respective substrate (Michel et al., 2006). However, these enzymes are not sufficient alone to lead to the complete substrate assimilation. As revealed by the increasing number of sequenced marine microbial genomes, marine bacteria possess a large number of sulfatases. Although their precise function has not been elucidated yet, it is likely that they play an important role in the degradation of algal sulfated polysaccharides (Glöckner et al., 2003).

While genomic and metagenomic approaches offer promising strategies for marine biodiscovery (Ekborg et al., 2006; Shin et al., 2010), there are still some limitations, as screening of such libraries is indeed either sequence based or function based (Kennedy et al., 2008). These limitations sometimes do not allow assigning new functions to proteins annotated as hypothetical, and in such cases, it is still necessary to go through the isolation and purification of a defined activity in order to ascribe a function to a gene.

As both exo- and endo-acting sulfatases have been demonstrated in the case of glycoaminoglycans, we postulate their existence in the case of carrageenans. We therefore screened marine bacteria for endo-sulfatases to specifically modify the sulfation pattern of carrageenans in a polymeric state. In a previous work, a carrageenan sulfatase converting ι- in α-carrageenan was isolated from *P. atlantica* T6C and recombinantly overexpressed (Préchoux et al., 2013). Analysis of its mode of action confirmed the endo-character of this sulfatase removing the sulfate ester groups, most likely in a random pattern along the polysaccharide chain. Aiming at monitoring the physico-chemical properties of carrageenan, we were looking for further sulfatases potentially having different properties as this would allow to fine tune the rheological properties of these hydrocolloids or to adapt to different industrial conditions. In this context, we isolated and purified to homogeneity an endo-sulfatase from the marine bacterium *P. carrageenovora* Psc^T^. Despite the fact that this enzyme has the same specificity as the ι-carrageenan sulfatase from *P. atlantica* (Préchoux et al., 2013), sequence analysis revealed that the *P. carrageenovora* sulfatase did not share any homology to already known sulfatases. It has, however, more than 90% sequence identity with a putative uncharacterized protein, Q3IKL4, from the marine strain *Pseudoalteromonas haloplanktis* TAC 125. Phylogenetic studies show that *P. carrageenovora* sulfatase thus represents the first characterized member of a new sulfatase family, with a C-terminal domain having strong similarity with the superfamily of amidohydrolases, highlighting the still unexplored diversity of marine polysaccharide modifying enzymes.

## Materials and methods

### Materials

λ-carrageenan (GENU X-7055) was extracted from tetrasporophytic plants of *Gigartina skottsbergii*, κ-carrageenan (GENU X-6913) was extracted from *Eucheuma cottonii* and ι-carrageenan (GENU X-6908) was extracted from *Eucheuma spinosum*. All of these samples were kindly provided by CP Kelco (Copenhagen). The marine bacteria *P. carrageenovora* strain Psc^T^ (ATCC 43555^T^) (Bellion et al., 1982) and *Pseudoalteromonas haloplanktis* 545^T^ (DSMZ 6060^T^) were obtained from the American Type Culture Collection and the Deutsche Sammlung von Mikroorganismen und Zellkulturen collection respectively.

### Production and purification of the ι-carrageenan sulfatase from *p. carrageenovora* (Psc ι-CgsA)

Unless otherwise stated, all purification steps were performed at 4°C. Hydrophobic interaction chromatography and anion exchange chromatography were carried out at 18°C. *P. carrageenovora* strain Psc^T^ was grown in 5 L of sulfate free ZoBell medium (ZoBell, 1941) containing 1g L^−1^ of λ-carrageenan. After 36 h of incubation at 15°C, the culture medium was centrifuged for 60 min at 1400–1800 *g*. The supernatant was discarded and the cells were slowly suspended for about 1 h in 30 mL of buffer A (50 mM Tris-HCl, pH 8.3) before lysis with a French Press. The lysate was centrifuged for 1 h at 29000 *g*. The cell-free supernatant was brought to 30% (NH_4_)_2_SO_4_ saturation (16.4 g (NH_4_)_2_SO_4_ per 100 mL of extract) by slow addition of (NH_4_)_2_SO_4_ salt. The sample was then centrifuged for 1 h at 25000 *g* and the pH of the supernatant was adjusted to 8.3 with 1 M Tris-HCl buffer before being loaded on a Phenyl Sepharose 6 fast flow high sub column (2.0 × 19.5 cm; GE Healthcare) equilibrated in buffer B (30% saturation (NH_4_)_2_SO_4_ in 50 mM Tris-HCl, pH 8.3). The stationary phase was washed with buffer B at a flow rate of 1.5 mL min^−1^ until the absorbance at 280 nm of the effluent was negligible. Elution of the bound proteins was then achieved at the same flow rate by applying a linear decreasing gradient from buffer B to buffer A during 20 column volumes (CVs). Fractions of 6.5 mL were collected.

The active fractions were pooled and dialyzed for 96 h against buffer A. The desalted sample (about 140 mL) was loaded at a flow rate of 2 mL min^−1^ on a Diethylaminoethyl (DEAE) Sepharose fast flow column (1.6 × 13 cm; GE Healthcare) equilibrated in buffer A. The column was washed at the same flow rate with buffer A until the absorbance measured at 280 nm was negligible. Elution of bound proteins was achieved at a flow rate of 2 mL min^−1^ with a linear increasing gradient from 0 to 1 M NaCl in buffer A. The final concentration of NaCl was reached after 20 CVs and 5.5 mL fractions were collected. The fractions containing pure *Psc* ι-CgsA were pooled and stored at 4°C in buffer A.

At the different steps of purification, the fractions were tested for sulfatase activity using standard ι-carrageenan as substrate. The standard reaction mixture contained 100 μL of protein fraction and 100 μL of 1.2% (*w/v*) ι-carrageenan both in 50 mM Tris-HCl buffer, pH 8.3. Incubation was performed for 12 h at 35°C. The reaction was stopped by diluting the incubation medium 2-fold and by centrifuging the samples in a Microcon-10 (Millipore) to remove the carrageenan. For each sample, the corresponding blank was performed in the same conditions but using the sample previously boiled for 10 min. The amount of free sulfate present in the filtrate was assayed by high pressure anion exchange chromatography (HPAEC) using a Dionex 500 chromatography system as described previously (Genicot-Joncour et al., 2009). Briefly, the anions present in the reaction medium were separated on an AS11 anion-exchange column (4 × 200 mm, Dionex) equipped with an AG11 guard column (4 × 50 mm, Dionex). Elution was performed with an isocratic gradient of 12 mM NaOH at a flow rate of 1 mL min^−1^ using a GP40 gradient pump (Dionex). Detection of the anions was carried out with an ED40 electrochemical detector in the conductivity mode. The peak of sulfate eluted separately from the other ions at 3 min and the concentration of sulfate was deduced from the signal intensity and calculated from a standard sulfate calibration curve. The active fractions were analyzed by SDS-PAGE (Laemmli and Favre, 1973) using 12% Criterion precast Bis-Tris gels (Bio-Rad). Gels were stained routinely with Coomassie blue R-250 and colloidal Coomassie blue staining when subjected to mass spectrometry analyses (Candiano et al., 2004). Protein quantification was performed according to Bradford (1976) using the Bio-Rad protein assay. Bovine serum albumin was used as a standard. Protein concentration of pure sulfatase was also estimated at 280 nm using a Nanodrop 2000 Spectrophotometer (Thermofisher). A molar extinction coefficient of 172.120 M^−1^ cm^−1^ and a molecular weight of 115.916 kDaltons (kDa), both deduced from the protein sequence (see below), were used to calculate the concentration of the enzyme.

### Protein sequence determination of the Psc ι-CgsA protein

The band corresponding to the *Psc* ι-CgsA sulfatase was excised from Coomassie blue stained SDS-PAGE and was subjected to in-gel tryptic digestion as described in Larré et al. (2010). Briefly, the gel slice was washed with 100 μL of 25 mM NH_4_HCO_3_, followed by dehydration with 100 μL of 50% (*v/v*) acetonitrile in 25 mM NH_4_HCO_3_. Proteins were reduced and alkylated by incubation for 1 h at 57°C in the presence of 10 mM Dithiothreitol (DTT), followed by 45 min of incubation at room temperature with 55 mM iodoacetamide. The gel slice was further washed with NH_4_HCO_3_ and dehydrated as described before. The band was then incubated overnight at 37°C with 10 μL of trypsin (sequencing grade, Promega) solubilized at 12.5 ng μL^−1^ in 25 mM NH_4_HCO_3_.The supernatant was collected and the tryptic fragments were analyzed by Matrix Assisted Laser Desorption Ionization coupled to a Time-of-Flight analyzer (MALDI-TOF) and nanoscale capillary liquid chromatography-tandem mass spectrometry (LC-MS/MS). MALDI-TOF mass spectrometry was performed on a M@LDI LR instrument (Waters). One microliter of the sample was mixed with 1 μL of the matrix preparation (2.5 g L^−1^ α-cyano-4-hydroxycinnamic, 2.5 g L^−1^ 2,5-dihydroxy benzoic acid, 70% (*v/v*) acetonitrile, and 0.1% (*w/v*) trifluoroacetic acid) and deposited onto the MALDI sample probe. Mass spectra were acquired on the mass-to-charge ratio range from 800 to 3000. LC-MS/MS analysis was performed using a nanoflow high pressure liquid chromatography (HPLC) system (Switchos-Ultimate II, Dionex) coupled to a hybrid quadrupole orthogonal acceleration time-of-flight mass spectrometer (Q-TOF Global, Waters). Chromatographic separations were conducted on a reverse-phase capillary column (75 μm i.d., Pepmap C18, Dionex) at a flow rate of 200 nL min^−1^ using a gradient from 2 to 50% of 0.08% (*w/v*) formic acid in acetonitrile. Mass data were recorded in “data dependent” mode: one MS spectrum was recorded on the mass-to-charge ratio range 400 to 1500 within 1 s, after which the three most intense ions were selected and fragmented in the collision cell. Raw data obtained by MALDI-TOF or LC-MS/MS were processed by means of the Protein Lynx Global Server v. 2.1. Software (Waters).

Protein identification was carried out by comparing the collected LC-MS/MS data against Uniprot databank. Databank searches were performed through the use of the Mascot server v. 2.2. program (Matrix Science). The mass tolerance was set to 120 parts per million (ppm) for parent ions (MS mode) and 0.3 Da for fragment ions (MS/MS mode), and one missed cut per peptide was allowed. Some MS/MS spectra were *de-novo* sequenced using the Protein Lynx Global Server v. 2.1. Software. This procedure was facilitated by the use of the OVNIp program (Tessier et al., 2010).

### Cloning, heterologous expression and purification of the Psc ι-CgsA

Genomic DNA from *P. carrageenovora* Psc^T^ was prepared as previously described (Barbeyron et al., 1984). The primers forward (5′-CCCCCCGAATTCTTATTTGTTGTTTTCAAAATAAAGTGGTTTAC-3′; *Eco*RI restriction site is underlined) and reverse (5′-GGGGGGGGATCCCAACAAGACGATGAGCCAAAATGG-3′; *Bam*HI restriction site is underlined), deduced from the gene PSHAa1171 of *P. haloplanktis* TAC 125 (Uniprot accession number Q3IKL4), were used to amplify the *Psc ι-cgsA* gene. The SignalP 3.0 program (Bendtsen et al., 2004) predicted the presence of a signal peptide with a cleavage site between residues Ala^22^ and Gln^23^ in the Q3IKL4 protein. Here, the gene without the signal peptide was cloned in between the *Bam*HI/*Eco*RI sites of the expression vector pFO4 (Groisillier et al., 2010) which encompass an N-terminal fused six-histidine-tag (6 His-tag). The sequence of the gene was checked using a genetic analyzer ABI 3130xl (Applied Biosystems) equipped with 50 cm capillaries and POP7^TM^ polymer. The amplified and verified gene sequence of *Psc ι-cgsA* was deposited at GenBank with accession number JN228253. The *Psc ι-cgsA* gene was also optimized for *Escherichia coli* codon use by GENEART (Life Technologies), amplified using the forward primer (5′-CCGGGGATCCCAGCAGGATGATGAACCG-3′; *Bam*HI restriction site is underlined) and the reverse primer (5′-GGCCGAATTCTTATTTGTTATTTTCAAAATACAG-3′; *Eco*RI restriction site is underlined) and cloned into the same expression vector.

For protein expression, transformed *E. coli* strains BL21(DE3) (Novagen^R^) were either grown at 20°C for 72 h in ZYP 5052 medium containing 200 μg. mL^−1^ ampicillin (Studier, 2005) or in Luria Bertani (LB) medium. In the latter case, the recombinant *E. coli* BL21 (DE3) cells were grown at 37°C in LB medium containing 100 μg.mL^−1^ ampicillin and 0.025% (*w/v*) glucose until the optical density at 600 nm reached ~1.2–1.5. The culture medium was then diluted twice with an equal volume of cold LB medium and buffered with HEPES buffer pH 7 to a 20 mM final concentration. Induction was performed by addition of lactose (0.6%) and isopropyl β-D-1-thiogalactopyranoside (IPTG) (2 mM) (Korf et al., 2005). The cultivation was further continued for ~18 h at 20°C until the optical density at 600 nm reached ~5–8. *E. coli* BL21 (DE3) bearing pFO4 without insert was used as the negative control. Culture was stopped by centrifugation at 1400–1800 *g* for 60 min. The pellet was then suspended in 50 mM Tris-HCl (pH 8.3) buffer containing 200 mM NaCl and 15 mM imidazole (buffer C) before lysis with a French Press. The lysate was centrifuged for 1 h at 29,000 *g*. The cell-free supernatant was then 0.2 μM filtered before being loaded onto a HisPrep FF 16/10 column (1.6 × 10 cm, GE Healthcare) equilibrated in buffer C. Elution of the protein was performed in buffer C using a linear gradient, increasing from 15 mM to 500 mM imidazole. The final concentration of imidazole was reached after 10 CVs and 2 mL fractions were collected. Fractions containing the recombinant tagged enzyme were estimated by SDS-PAGE analysis and by Western blot. Transfer from SDS gel onto ready to use 0.2 μm nitrocellulose membrane (BioRad) was performed using a Trans Blot Turbo system in the conditions specified by the manufacturer (BioRad). Monoclonal anti-polyhistidine peroxidase conjugate (Sigma) was used at a final concentration of 1/10,000 to specifically recognize the his-tagged fusion proteins. Immuno-detection was performed by chemiluminescence using the Clarity Western ECL Substrate kit (BioRad) and visualization was achieved using the Chemi-Capt 50001 software. Recombinant enzyme activity was tested using 4- methylumbeliferyl sulfate (potassium salt, Sigma), further on called MUFS, as substrate. Unless explicitly indicated in the text, the standard conditions included 8–20 μg sulfatase, 50 mM Tris-HCl pH 8.3, 200 mM NaCl, 850 μM MUFS in a 150 μl reaction volume. The reaction was carried out for up to 120 min at 35°C and the increase of absorbance was measured as a function of time at 360 nm using a Saphire2 microplate reader (Tecan, Männedorf, Switzerland). For each reaction, a blank was made using the negative control at the same protein concentration than that of the sample.

### Biochemical characterization of the native Psc ι-CgsA

Sulfatase activity of the native enzyme was characterized using ι-carrageenan as substrate and the amount of sulfate released was determined by HPAEC as described above. pH optimum determination was performed at 35°C in a pH range of 5.8–9.5. Assays were carried out by incubating 50 μl of ι-carrageenan (1.8% *w/v* in water) with 80 μl of 500 mM buffer and 40 μl of sulfatase solution (600 μg mL^−1^ in 50 mM Tris-HCl pH8). The solutions used to buffer the reactions were as follows: 500 mM 2-(*N-morpholino)* ethanesulfonic acid (MES) (pH 5.5 to pH 6.9), 500 mM Bis-Tris Propane (pH 6.5 to pH 9.5) and 500 mM Tris-HCl (pH 7 to pH 9). The optimal temperature was determined by measuring the amount of sulfate released upon incubation of 35 μl of sulfatase solution (600 μg mL^−1^) with 115 μl of ι-carrageenan (0.6%, *w/v*) both in 50 mM Tris-HCl pH 8.0 in a range of 5–55°C in steps of 5°C. The effect of ionic strength on enzyme activity was assessed using increasing NaCl concentration from 0 to 500 mM. The influence of phenylmethylsulfonyl fluoride (PMSF), magnesium chloride (MgCl_2_), and DTT on the sulfatase activity was tested at final concentrations of 1 and 2 mM, whilst chelators agents were tested at final concentrations ranging from 2 to 10 mM. All these assays were performed at 30°C by incubating 50 μl of ι-carrageenan (1.5% *w/v in* 50 mM Tris-HCl pH8) with 20 μl of sulfatase solution (670 μg mL^−1^) in 120 μl total volume The relative activity was defined as the percentage of the activity observed without addition of these reagents.

### Biochemical characterization of the recombinant sulfatase

Sulfatase activity of the recombinant enzyme was characterized using MUFS in the standard conditions described above. The influence of pH on the activity was determined using the same buffers as for the native enzyme. The effect of different additives on the recombinant enzyme activity was studied in the same concentration conditions as those used for the characterization of the native enzyme. However, prior to the determination of the effect of ionic strength on the enzyme activity, the sample of recombinant enzyme was dialyzed overnight against 50 mM Tris-HCl pH8.3. In the same way as for the native enzyme, the relative activity was defined as the percentage of the activity observed without addition of these reagents.

### Kinetic parameters of native and recombinant Psc ι-CgsA

As the ι-carrageenan is viscous even at low concentration, it was not possible to prepare a sufficient range of ι-carrageenan concentrations to allow for the accurate determination of kinetic parameters of the enzymes. For this reason, the Michaelis parameters of both enzymes, purified from *P. carrageenovora* culture medium and recombinantly expressed in *E. coli*, were determined at optimum conditions of the native enzyme, using MUFS as substrate. Kinetic measurements were conducted in 150 μl final volume and 80 μl of sulfatase were incubated with 2 μl of MUFS at final concentrations ranging from 5.2 to 206.5 μM. The formation of methylumbelliferone was measured as a function of time at 360 nm (molar extinction coefficient of 5143 M^−1^ cm^−1^) (Viladot et al., 1998), using a Saphire2 microplate reader (Tecan, Männedorf, Switzerland). The experiments were carried out in triplicates. Kinetic experiments were performed for up to 120 min at 35°C. The initial velocities were measured on the linear section of the kinetics plots and the apparent K_m_ and V_max_ were calculated from an hyperbolic regression analysis using the software Hyper32 version 1.0.0. (http://homepage.ntlworld.com/john.easterby).

### Nuclear magnetic resonance (NMR) spectroscopy

The ι-carrageenan incubated with pure native *Psc* ι-CgsA was freeze dried, exchanged twice with 99.97 atom % ^2^H_2_O and then dissolved in ^2^H_2_O at a final concentration of 10 mg mL^−1^. The product was then transferred into a 5 mm NMR tube and ^1^H-NMR spectra were recorded at 70°C, using a BRUKER Advance DRX 500 spectrometer equipped with an indirect 5 mm gradient probehead TXI ^1^H/^13^C/^31^P. Chemical shifts are expressed in ppm in reference to trimethylsilylpropionic acid (TSP), which was used as an external standard. No suppression of the HOD signal was performed.

### Sequence and phylogenetic analysis

Searches for protein sequence similarities were performed in Uniprot database using the BlastP program (Altschul et al., 1997). Protein structure prediction was performed with the Phyre2 server (Kelley and Sternberg, 2009). A multiple alignment was generated with 421 sequences using the MAFFT program and L-INS-i algorithm (Katoh et al., 2005) and manually refined. From the multiple alignments, 171 positions were used to build a phylogenetic tree by the Maximum Likelihood method using the Whelan and Goldman evolution model (Whelan and Goldman, 2001). Reconstruction of the tree and bootstrap analysis (resampling of 100) were conducted with the MEGA v. 5.05. Software (Tamura et al., 2011).

## Results

### Screening and purification of the Psc ι-CgsA

The marine bacterium *P. carrageenovora* was screened for potential sulfatase activity capable to catalyze the hydrolysis of sulfate from carrageenans. As a consequence the use of carrageenans as substrate was mandatory during the screening and the sulfate assay was performed by HPAEC as described previously (Genicot-Joncour et al., 2009). To avoid interference of ions with the detection of sulfate groups during chromatography analysis, *P. carrageenovora* strain Psc^T^ was grown in ZoBell medium.

Carrageenan-sulfatase activities were detected only when the culture medium was supplemented with κ-, ι- or λ-carrageenan. As reported in Table 1, carrageenan-sulfatase activities were measured both in the bacterial pellet and in the culture supernatant. ι- and λ-carrageenan proved to be good inducers of carrageenan-sulfatases that were active on κ-, ι- and λ-carrageenan. In this study, we focus our attention on the ι-carrageenan sulfatase activity detected in the pellet of *P. carrageenovora* cultures induced by the λ-carrageenan.

**Table 1:** **Sulfatase activity in cultures of *P. carrageenovora* Psc^T^ and *P. haloplanktis* 545^T^**.

	**Inducer**	**Total Proteins**	**Substrate**		
			**κ-carrageenan**	**ι-carrageenan**	**λ-carrageenan**
Supernatant	κ-carrageenan	1.6 (1.1)	n.d. (1.5)	n.d. (13.4)	0.6 (n.d.)
	ι-carrageenan	1.6 (0.5)	6.3 (n.d.)	11.9 (n.d.)	n.d. (n.d.)
	λ-carrageenan	2.35 (0.8)	n.d. (n.d.)	n.d. (73.2)	4.5 (7.7)
Pellet	κ-carrageenan	6.5 (4.1)	0.9 (n.d.)	n.d. (n.d.)	n.d. (n.d.)
	ι-carrageenan	10.5 (3.8)	4.0 (n.d.)	4.7 (n.d.)	n.d. (n.d.)
	λ-carrageenan	30 (5.9)	2.1 (n.d.)	10.4 (n.d.)	0.4 (n.d.)

Cultures of P. carrageenovora Psc^T^ and P. haloplanktis 545^T^ were induced with 0.1% (w/v) κ-, ι-, or λ-carrageenan. Incubations were performed for 12 h as described in material and methods using κ-, ι- or λ-carrageenan as substrate. Activities are expressed in nmole of sulfate released per minute and per mg of total proteins (nmole min^−1^ mg^−1^). The amount of total proteins are expressed in mg. Data in brackets are relative to Pseudoalteromonas haloplanktis 545^T^. n.d. means that no activity was detected.

**Figure 2:**
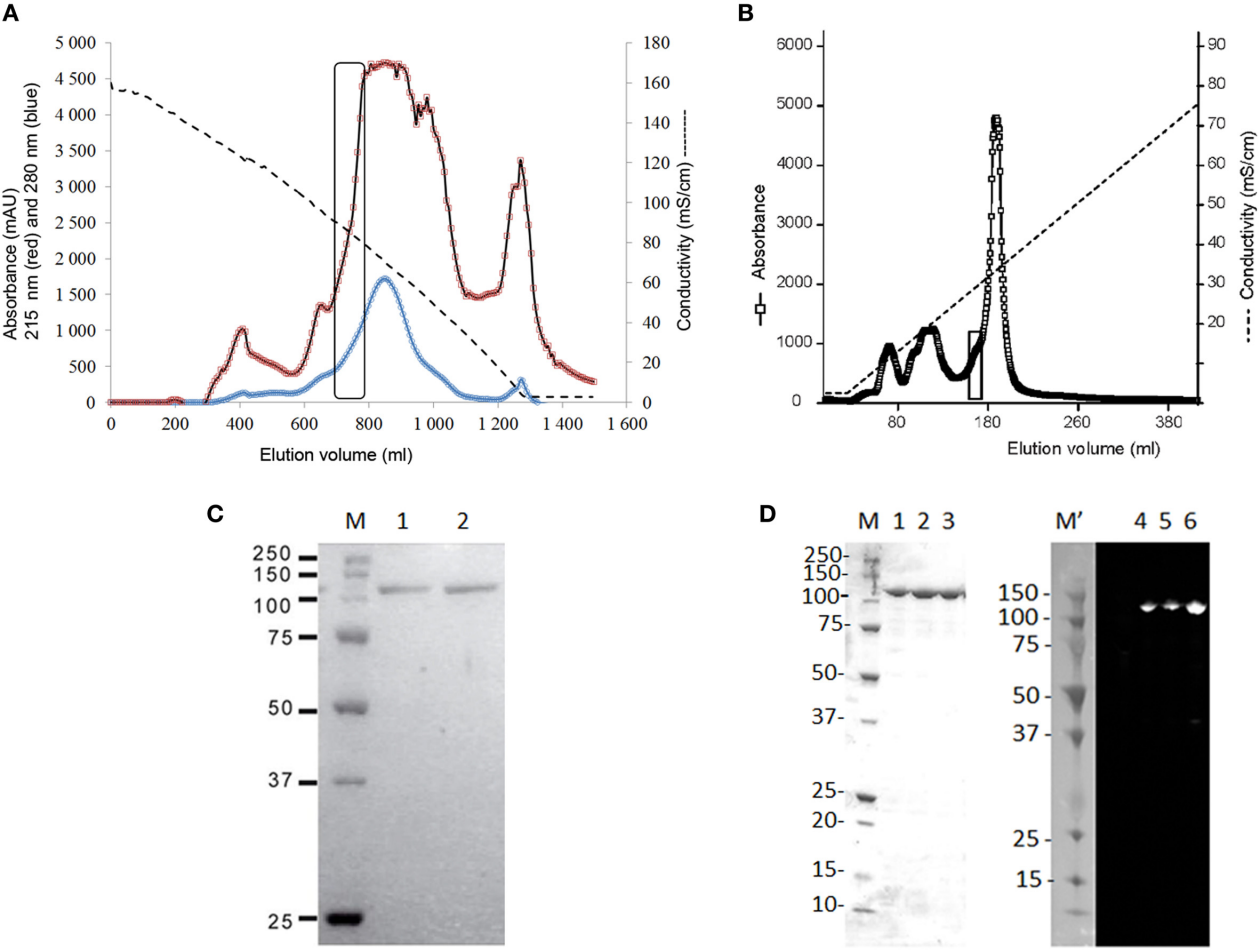
**Purification of the ι-carrageenan sulfatase. (A)** Purification of the native ι-carrageenan sulfatase on a Phenyl Sepharose 6 fast flow high sub column (2.0 × 19.5 cm). Elution of proteins was performed using a decreasing gradient from 30% saturation (NH_4_)_2_SO_4_ in buffer A to 0% (dashed lines). The absorbance of the fractions (6.5 ml) was measured both at 280 nm (blue curve) and at 215 nm (red curve). Active fractions containing sulfatase activity are surrounded with a rectangle. They were pooled and dialyzed against buffer A prior being loaded on top of the anion exchange chromatography, as described in material and methods. **(B)** Purification of the native ι-carrageenan sulfatase on a DEAE-Sepharose fast flow column (1.6 × 13 cm). Elution of proteins from the anion exchange chromatography was performed using an increasing gradient from 0 to 1 M NaCl (dashed line). The absorbance of the elution fractions was measured at 215 nm (open squares). Fractions containing pure *Psc* ι-CgsA are surrounded with a rectangle. **(C)** SDS- PAGE analysis of the native ι-carrageenan sulfatase isolated from *P. carrageenovora*. Elution fractions from the DEAE Sepharose chromatography were separated on 12% SDS-PAGE and stained with Colloïdal Coomassie Blue (Lanes 1–2). Precision protein standards from Bio-Rad were used as markers (Lane M, from top to bottom: 250 kDa, 150 kDa, 100 kDa, 75 kDa, 50 kDa, 37 kDa, and 25 kDa). **(D)** SDS- PAGE and Western Blot analysis of the recombinant his-tagged ι-carrageenan sulfatase after affinity chromatography. Lane M corresponds to the molecular weight markers (as above and in addition 20 kDa, 15 kDa, and 10 kDa). Lanes 1 to 3: elution fractions from the HisPrep chromatography. Precision Plus protein dual color standards from BioRad were used as makers for the Western blot (Lane M', sizes same as lane M). Lanes 4, 5, and 6 correspond to the Western blot analysis of the his-tagged sulfatase. Revelation was performed by chemiluminescence as described in material and methods.

The newly detected enzyme, further on named *Psc* ι-CgsA, was purified by a combination of ammonium sulfate fractionation, hydrophobic interaction chromatography and ion exchange chromatography. The first chromatographic step using hydrophobic interaction chromatography on phenyl-Sepharose was efficient in purifying the sulfatase as this one elutes between 91 and 78 mS cm^−1^, just before the majority of the proteins (Figure 2A). All the active fractions were pooled and dialyzed before being loaded on top of the anion exchange chromatography column (DEAE Sepharose). At this stage, the sulfatase eluted between 300 and 370 mM NaCl i.e., 29.5 and 32.3 mS cm^−1^ respectively (Figure 2B). The purified enzyme gave a single band with an apparent molecular mass of 110 kDa in SDS-PAGE under reducing conditions (Figure 2C).

### Identification of the Psc ι-CgsA protein, a novel sulfatase

**Figure 3:**
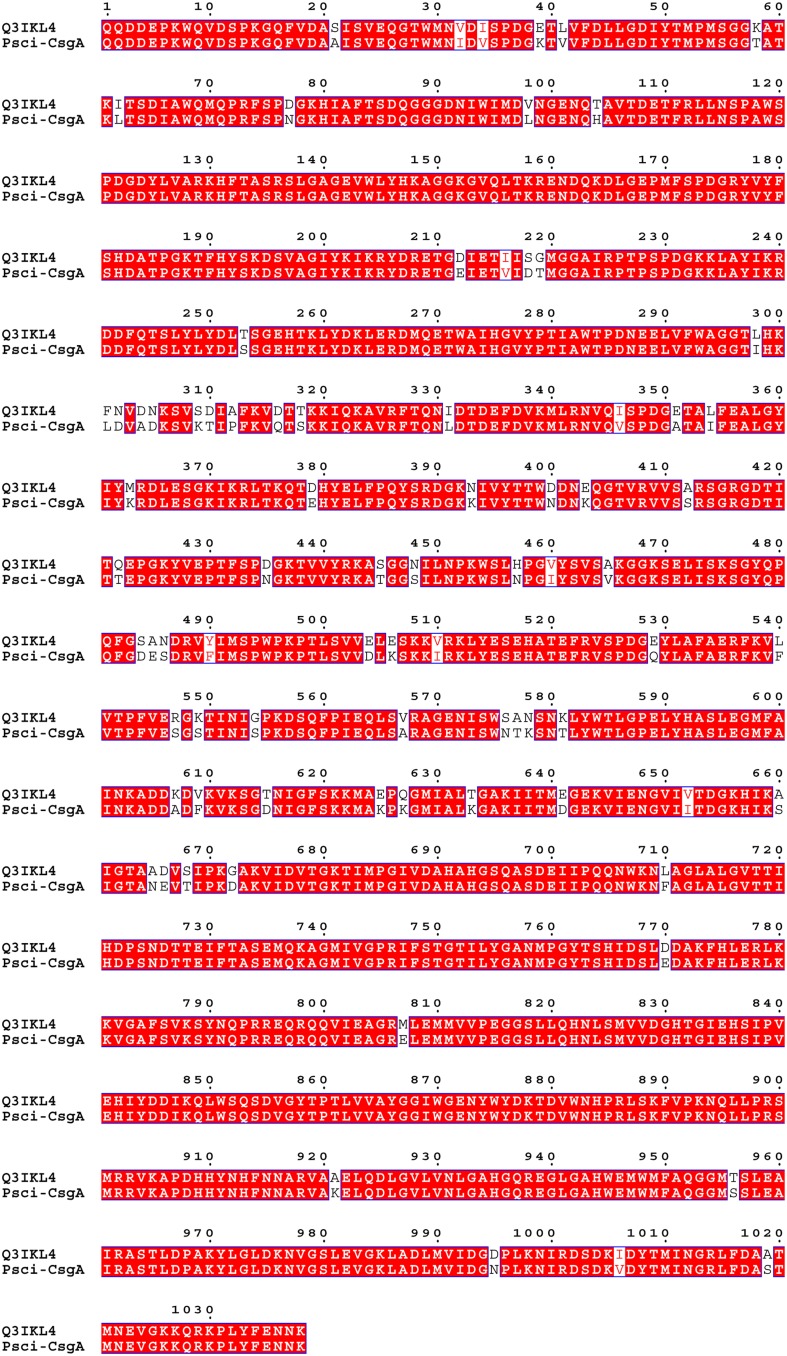
**Sequence alignment of *Psc* ι-CgsA and Q3IKL4_PSEHT from *P. haloplanktis* TAC 125 using the program MEGA 5**. The ESPript 3 program (Robert and Gouet, 2014) was used to enhance the conserved amino acids which are shown in white letters on a red background.

Following digestion of the pure *Psc* ι-CgsA protein by trypsin, the peptide fragments were analyzed by LC-MS/MS and compared to the Uniprot database. Eight peptides (YVEPTFSPDGK, VIENGVIITDGK, DLGEPMFSPDGR, SLGAGEVWLYHK, YVYFSHDATPGK, FTQNLDTDEFDVK, LLNSPAWSPDGDYLVAR, VSPDGQYLAFAER) were identical to peptides found in a putative uncharacterized protein Q3IKL4 (117.873 kDa), encoded in the genome of the marine strain *P. haloplanktis* TAC 125 (Medigue et al., 2005). In depth sequencing by MALDI-MS and by *de novo* allowed to cover 14 additional sequence stretches (LYESEHATEFR, QQVIEAGR, TDVWNHPR, AGENLS, LVYTTW, VLDG-PL, WSLNPG-YSV, LTSDLA—QPR, QPQFG—DR, YELF-QYSR, ELF-QYSR, VTPFVE—–LNSP, LF-A-TM–VGKK, ELETVL). Altogether, peptide sequencing of the *Psc* ι-CgsA provided coverage of 23% of the 1060 amino acids of the *P. haloplanktis* Q3IKL4 protein (Figure 3). The sequence of the here described novel *Psc* ι-CgsA was further confirmed as described in the following section.

### Sequence determination, cloning and overexpression of the ι-CgsA sulfatase

The nucleotide sequence encoding the Q3IKL4 protein without the peptide signal was used to design specific oligonucleotide primers that were used to clone and sequence the gene encoding the *Psc* ι-CgsA *(ι-cgsA*) from *P. carrageenovora*. After successful expression and sequencing, the translation of the *ι-cgsA* gene yielded a protein of 1037 amino acids (GenBank accession number JN228253) with a theoretical isoelectric point (pI) value of 6.78 and a molecular weight of 115.9 kDa. This mass corresponds to the apparent molecular mass of the purified native and expressed *Psc* ι-CgsA (~110 kDa), deduced from SDS-PAGE. As expected, all the peptide sequences identified by mass spectrometry analysis were present in the protein sequence. The *Psc* ι-CgsA shares 92.6% sequence identity with the putative uncharacterized protein Q3IKL4 of *P. haloplanktis* TAC 125 (Figure 3).

*Psc* ι-CgsA was expressed as a soluble protein at about 6.5 mg L^−1^ in *E. coli* BL21 (DE3) when induced with 0.2% lactose (ZYP 5052 medium). The yield was more than doubled (16.9 mg L^−1^) when induction was carried out with both lactose (0.6%) and IPTG (2 mM). After affinity chromatography on a HisPrep sepharose column, the enzyme was pure (Figure 2D). As shown on Figure 2D, the use of anti-histidine antibody allows recognizing the his-tagged protein which migrates as a single band at the expected size.

### Biochemical characterization of the native Psc ι-CgsA protein

The purified sulfatase was kinetically evaluated using carrageenan as substrates. The amount of sulfate released after incubation of the pure sulfatase using ι-, κ- or λ-carrageenan was monitored by HPAEC. In the presence of κ- and λ-carrageenan, no desulfation was observed, even after prolonged incubation. In contrast, strong desulfation occurred when ι-carrageenan or hybrid ι-carrageenan containing about 20% of ν-carrabiose was used as substrate, highlighting the specificity of this sulfatase toward the ι-carrabiose moiety.

**Figure 4:**
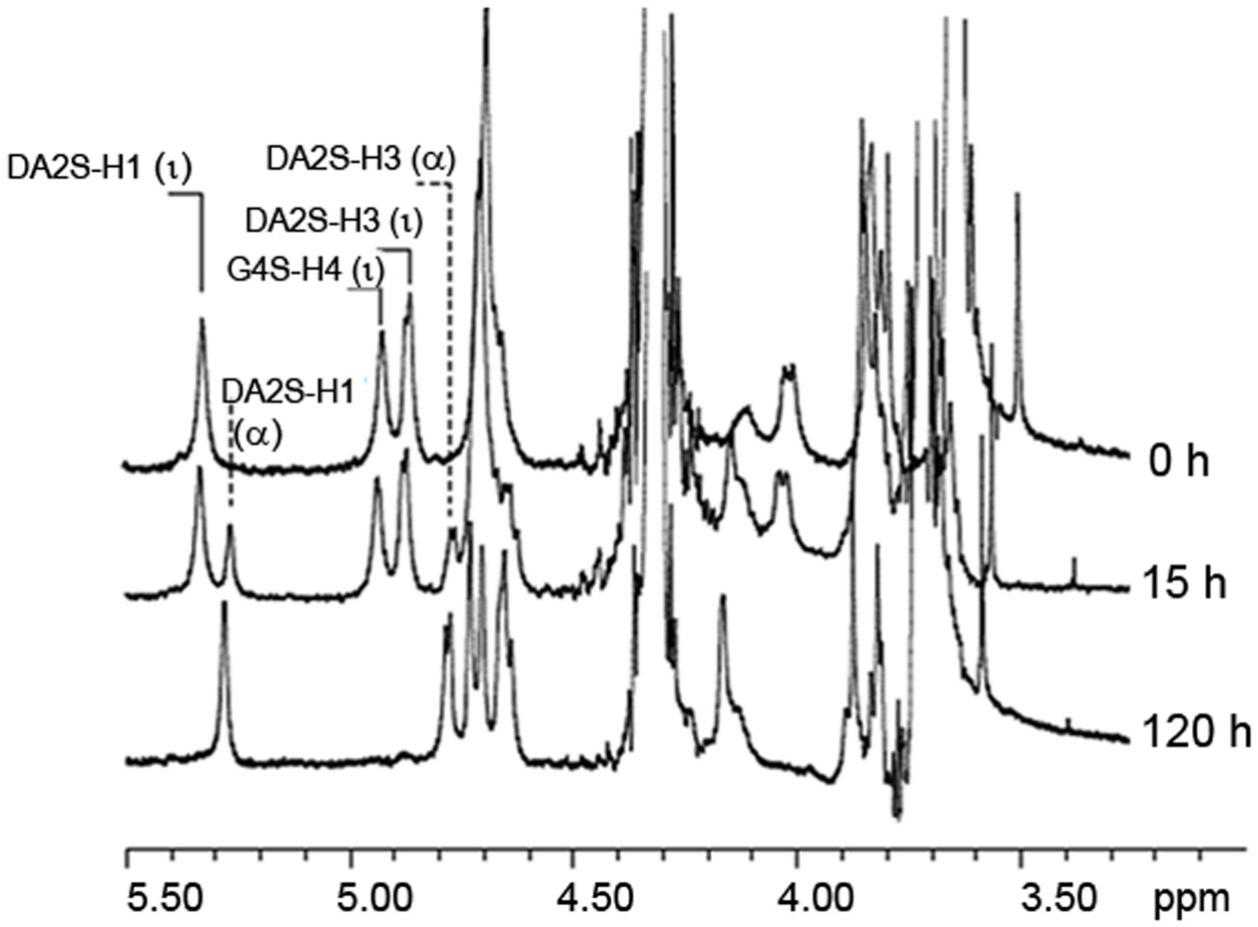
**ι-carrageenan desulfation with pure native ι-carrageenan sulfatase**. ^1^HNMR spectra monitoring the bioconversion of standard ι-carrageenan (Top spectrum, 0 h) into α-carrageenan (Bottom spectrum). This conversion was performed after 120 h of incubation of ι-carrageenan with pure *Psc* ι-CgsA (Bottom spectrum, 120 h). Intermediate structures observed after 15 h of incubation (Middle spectrum, 15 h), are hybrid ι-/α-carrageenans whose composition was deduced by integrating the α-anomeric signal of DA2S-H1 (ι) or (α) indicated on the spectra (Falshaw et al., 1996; van de Velde et al., 2002).

As shown in Figure 4, the desulfation reaction of ι-carrageenan by the Psc-ι sulfatase was slow, in the range of hours. The rate of sulfate release increased with the concentration of ι-carrageenan up to 0.6% (*w/v*). At higher concentrations the medium became very viscous interfering with the enzyme diffusion. When 0.5% (*w/v*) ι-carrageenan was used, the rate of desulfation was linear for the first 15 h. Under these experimental conditions the temperature optimum was determined to be 40 ± 5°C and the pH optimum was measured at 8.3 (Table 2). The *Psc* ι-CgsA could be kept for several months at 4°C at this pH. Addition of sodium chloride at high concentrations affects the enzyme activity. Indeed, at 500 mM NaCl, the activity drops to 17.5% of the optimal activity, observed at 200 mM NaCl (Table 2).

**Table 2:** **Biochemical characterization of the native *Psc* ι-CgsA compared to two other polysaccharide sulfatases described in the literature**.

**Characteristics**	**Native *Psc* ι-CgsA**	***P. atlantica* sulfatase**	***P. carrageenovora* arylsulfatase**
Sulfatase family	New sulfatase family	Formylglycine family	β- lactamase superfamily New sulfatase family
Polysaccharide substrate	ι-carrageenan	ι-carrageenan	Agar
Molecular mass	115.9 kDa	55.7 kDa	35.8 kDa
Theoretical pI	6.8	7.1	5.4
Optimal temperature	35–45°C	35°C	45°C
Optimal pH	8.3	7.5	7.0–8.5
Optimal (NaCl]	200 mM	~25 mM	~500 mM
EDTA 1 mM	0% inhibition	Data not available	53.2% inhibition
PMSF 1 mM	91% inhibition	Data not available	26% inhibition
Phosphate buffer	82% inhibition	Data not available	Inhibition

Both the native *Psc* ι-CgsA and the ι-carrageenan sulfatase from *P. atlantica* (Préchoux et al., 2013) were characterized using ι-carrageenan as substrate. The data are compared to those of the arylsulfatase isolated from *P. carrageenovora* (Barbeyron et al., 1995; Lim et al., 2004; Kim et al., 2005).

With the aim to determine the specificity of the ι-carrageenan sulfatase more precisely, the structural modification of ι-carrageenan was monitored, as a function of time, by ^1^H NMR (Figure 4). The signals of ^1^H NMR spectrum of the standard ι-carrageenan (Figure 4 top spectrum: 0 h) were attributed on the basis of previously reported analyses (van de Velde et al., 2002). The signal observed at 5.32 ppm corresponds to the anomeric proton of the 4-linked anhydrogalactose unit (DA2S-H1). The G4S-H4 and DA2S-H3 protons, which signals are at 4.91 ppm and 4.85 ppm respectively, are also indicated on the spectrum. These signals decreased after 15 h of incubation of 41.25 mg of ι-carrageenan with 1.12 mg of ι-CgsA whilst two other signals, at 5.26 ppm and 4.80 ppm, appeared (Figure 4, middle spectrum: 15 h). To overcome heat inactivation of the enzyme, 300 μg of *Psc* ι-CgsA were added after 60 h. This addition, combined with prolonged enzymatic incubation (up to 120 h), allowed the complete modification of the ι-carrageenan (Figure 4, bottom spectrum: 120 h). Based on the analyses reported by Falshaw et al. (1996), this spectrum is characteristic of alpha (α)-carrageenan. Signals assignable to κ- (5.1 ppm) or beta (β)-carrabiose units (5.09 ppm) or to the production of reducing and non-reducing ends were not observed. This suggests that *Psc* ι-CgsA specifically catalyzes the conversion of ι-carrabiose into α-carrabiose units (Figure 1) within a polysaccharide chain and is devoid of glycoside hydrolase activity.

### Biochemical characterization of the recombinant ι-CgsA protein

The purified recombinant ι-CgsA was able to specifically remove sulfate from ι-carrageenan. After 24 h of incubation at 35°C and pH 8.3, the amount of sulfate released represents roughly 3% of the total sulfate of the polymer. Although this is not sufficient to detect the conversion of ι-carrageenan into α-carrageenan by ^1^H NMR, this low but significant activity confirmed that the product of the cloned gene was a ι-carrageenan sulfatase. As a consequence of the low activity, all further characterization of the recombinant enzyme was performed using an artificial substrate which is MUFS. As shown on Table 3A, both native and recombinant enzymes are most active at 35°C and pH 8.3. Although the recombinant sulfatase shows an activity in MES comparable to that observed in the Tris buffer, its activity drops drastically in the 3-morpholinopropane-1-sulfonic acid (MOPS) buffer and in the phosphate/citrate buffer deviating from the trend of pH profile (data not shown). This therefore suggests that, in the case of the later buffer, inorganic phosphate may be an inhibitor of the ι-sulfatase.

**Table 3:** **Characterization of the native and the recombinant *Psc* ι-CgsA**.

**(A) BIOCHEMICAL CHARACTERIZATION**		
**Characteristics**	**Native *Psc* ι-CgsA**	***Recombinant Psc* ι-CgsA**
Optimal temperature (°C)	35–45	35
Optimal pH	8.3	8.4 ± 0.3
Optimal [NaCl] (mM)	200	100
EDTA 10 mM	132.8% ± 12.9	93.6% ± 3.3
EGTA 10 mM	125.5% ± 9.2	100.9% ± 8.5
MgCl_2_ up to 2 mM	37.8% ± 2.8	81.7% ± 2.5
DTT 1 mM	46.8% ± 4.2	74.8% ± 6.9
PMSF 2 mM	0%	34.1% ± 13.4

Characterization of the native enzyme has been performed using ι- carrageenan whereas MUFS was used for the recombinant enzyme. As stated in material and methods, incubation with ι- carrageenan was performed during 12 h whilst kinetics with MUFS were carried out for 2 h. Unless otherwise stated, the activity is expressed as the percentage of the initial activity observed without addition of reagents.

**Table:** 

(B) KINETIC PARAMETERS			
Enzyme	Apparent Km (μM)	*kcat* min^−1^	*Kcat*/Km (μM^−1^ min^−1^)
Native *Psc* ι-CgsA	21.9 ± 8.2	6.32 ± 0.38	0.280 ± 0.05
Recombinant ι-CgsA	13.4 ± 4.9	1.64 ± 0.23	0.12 ± 0.04
Arylsulfatase from *P. carrageenovora*	68	ND	ND

Preliminary kinetic parameters of the native and recombinant *Psc* ι-CgsA were measured using the optimal reaction conditions of the enzymes as determined above and the artificial substrate MUFS at concentrations ranging from 5.2 to 206.5 μM. Data are compared to that of the *P. carrageenovora* arylsulfatase (Barbeyron et al., 1995) using the same substrate. N.D. means not determined.

The activity of the recombinant ι-CgsA was investigated in presence of different additives (Table 3A), to be compared to the native enzyme. Among them, Mg^2+^ and DTT inhibit slightly the enzyme whilst the addition of chelating agents such as EDTA an EGTA at 2 mM does not affect the activity. EDTA has to be used at a concentration of 10 mM to induce a slight decrease of the activity. The recombinant enzyme is completely inhibited when the concentration of NaCl reaches 500 mM. At 200 mM NaCl, the recombinant enzyme has 89.6% of its optimal activity observed at 100 mM NaCl.

**Figure 5:**
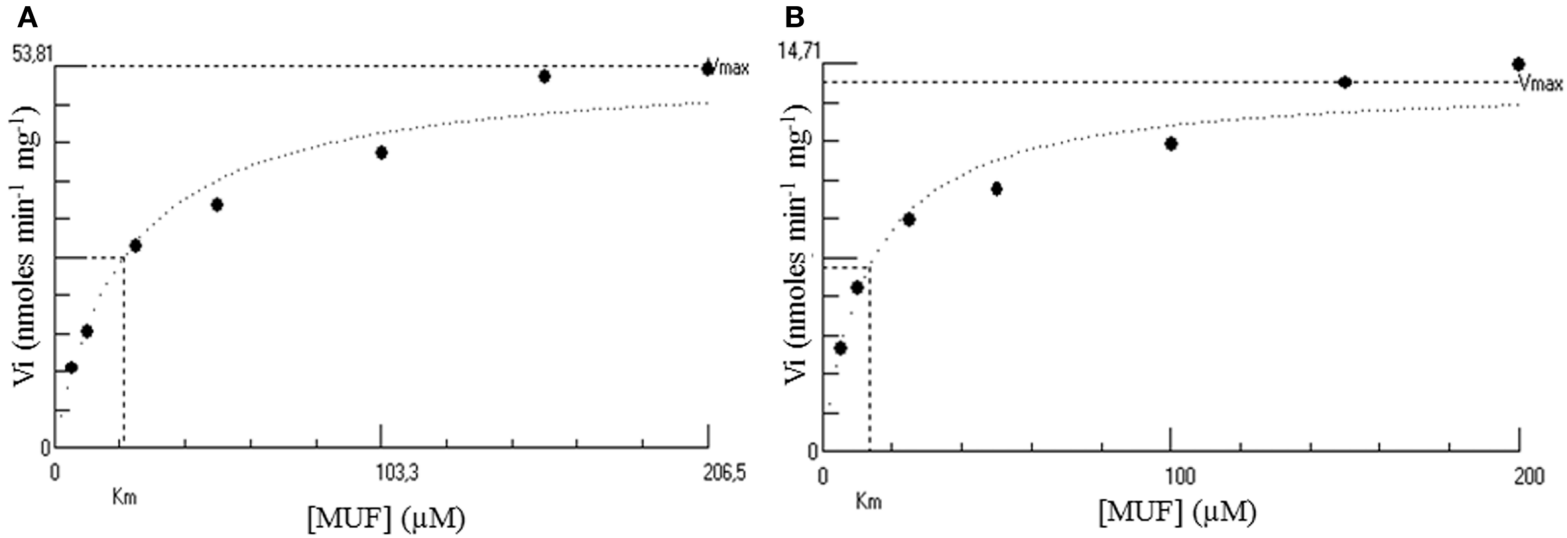
**Hyperbolic regression determination of the kinetic parameters of both the native (A) and the His-tagged (B) *Psc* ι-CgsA**. Kinetics were performed at 35°C using MUFS at different concentrations from 5.2 to 206.5 μM. These plots have been performed using the software Hyper32 version 1.0.0. (http://homepage.ntlworld.com/john.easterby). They are representative of experiments performed in triplicates.

The Michaelis parameters of both enzymes using an artificial substrate have been determined in optimal conditions using MUFS as substrate (Figure 5). These preliminary experiments show that while the K_m_ of both enzymes are in the same order of magnitude, the *kcat* of the native enzyme is about 3 times that of the recombinant enzyme (Table 3B).

## Discussion

### Discovery of a ι-carrageenan sulfatase, ι-CgsA, specifically releasing sulfate from the d-galactose-4-sulfate units in ι-carrageenan

Many genes encoding sulfatases have been cloned from all kingdoms of life (Sasaki et al., 1988; de Hostos et al., 1989; Paietta, 1989; Yang et al., 1989; Hallmann and Sumper, 1994; Ferrante et al., 2002; Medigue et al., 2005; Sardiello et al., 2005; Frese et al., 2008), but the number of characterized sulfatases remains limited and does not reflect the huge chemical diversity of sulfated biomolecules. The recent discovery that a vast number of sulfatases are present in marine bacteria (Glöckner et al., 2003; Barbeyron, personal communication) highlights the untapped resource of this type of activity in the marine environment. Indeed, most of the characterized sulfatases are specific of metabolizing glycosaminoglycans (Buono and Cosma, 2010), due to their importance in pathogenicity or human health. Nevertheless, it has been shown that the sulfatase from *Sphingomonas* sp. AS6330 (Kim et al., 2004) and the arylsufatase AtsA from *P. carrageenovora* Psc^T^ (Lim et al., 2004; Kim et al., 2005) catalyze the desulfation of agar. Recently, a formylglycine-dependent endo-4S-ι-carrageenan sulfatase from the marine bacterium *P. atlantica* T6c has been purified and characterized (Préchoux et al., 2013). In the present study we have identified, sequenced and characterized a novel ι-carrageenan sulfatase, *Psc* ι-CgsA, first member of a new family of sulfatases. NMR studies of the reaction products unambiguously demonstrate that *Psc* ι-CgsA catalyzes the removal of the sulfate ester group localized on the position C4 of the G4S moieties from the ι-carrabiose units, leading to the formation of α-carrabiose units. Despite the complete absence of sequence homology, this activity appears to be the same as that already described for the formylglycine-dependent sulfatase from the marine bacterium *P. atlantica* T6c, indicating that this catabolic activity plays an important role in carrageenan metabolization.The desulfation is ι-carrageenan specific, since neither the *P. atlantica* 4S-ι-carrageenan sulfatase nor the here described *Psc* ι-CgsA sulfatase are able to catalyze the removal of the 4S sulfate group from κ-carrabiose units. In κ-carrageenan, the G4S residues are located between two neutral anhydrogalactose residues whilst in ι-carrageenan the G4S moieties are positioned between two sulfated anhydrogalactose residues (Figure 1). This finding therefore allows the assumption that the substrate specificity in these endo-sulfatases includes the recognition of at least one additional sulfated anhydrogalactose in their substrate binding site to display the observed specificity.

### The ι-carrageenan sulfatase ι-CgsA is a modular enzyme belonging to a novel class of sulfatases

Despite their identical substrate specificity, the ι-carrageenan sulfatases of *P. carrageenovora* Psc^T^ and *P. atlantica* T6c have very different biochemical characteristics (Table 2) and can also be distinguished by their primary sequence that do not share enough similarity to allow an accurate sequence alignment. The ι-carrageennan sulfatase from *P. atlantica* T6c is indeed a formylglycine sulfatase and in this respect it has the characteristic signatures of family 1 formylglycine-dependent sulfatases, namely [SAPG]-[LIVMST]-[CS]-[STACG]-P-[STA]-R-x(2)-[LIVMFW](2)-[TAR]-G and G-[YV]-x-[ST]-x(2)-[IVAS]-G-K-x(0,1)-[FYWMK]-[HL] (Prosite signatures PS00523 and PS00149 respectively), which are well conserved in this family of enzymes. These signatures are not present in the *Psc* ι-CgsA sulfatase, thus indicating that this latter enzyme does not belong to the well-defined and represented family of formylglycine-dependent sulfatases. Sequence alignment of the Psc-ι sulfatase with the protein Q3IKL4 from *P. haloplanktis* and 9 other proteins chosen amongst the ones representing the new sulfatase family revealed that no cystein amino acids are conserved (Supplementary Figure 1). However, several conserved serine could potentially be subjected to post-translational modification. Therefore, and based on sequence data alone, it cannot be completely excluded (although improbable) that this sulfatase potentially reveals the existence of a new formylglycine-dependent family. It would however involve a serine modification. Interestingly, a ι-carrageenan sulfatase was revealed to be very active in *P. haloplanktis* 545^T^ extracts. This enzyme was induced in similar conditions to those observed for induction of the *Psc* ι-CgsA (Table 1, data in brackets). Since the genomes of both sequenced strains of *P. haloplanktis* TAC 125 (Medigue et al., 2005) and *P. haloplanktis* ANT/505 (GenBank ADOP00000000.1) do not contain any formylglycine-dependent sulfatase genes, it is tempting to assume that the sulfatase activity detected in *P. haloplanktis* 545^T^ is due to a ι-CgsA-type enzyme, such as in *P. carrageenovora*. Indeed, as described above, proteins orthologous to *Psc* ι-CgsA are present both in *P. haloplanktis* TAC 125 and ANT/505.

A BlastP sequence similarity search with the *Psc* ι-CgsA used as query sequence against the Uniprot database revealed that *Psc* ι-CgsA showed more than 90% identity with putative uncharacterized proteins from several marine species of *Pseudoalteromonas*. Moreover, many proteins from different marine strains such as *Colwellia psychrerythraea* 34H (Methe et al., 2005) and *Shewanella sediminis* HAW-EB3, belonging to the amidohydrolase superfamily, exhibited more than 60% of identity with the *Psc* ι-CgsA sulfatase (Supplementary Figure 1). Three-dimensional structure modeling of *Psc* ι-CgsA using the Phyre2 (Kelley and Sternberg, 2009) tool reveals that *Psc* ι-CgsA most likely has a multi-modular arrangement that covers 94% of the residues and are modeled with more than 90% of confidence. From this model it is hypothesized that *Psc* ι-CgsA consists of an N-terminal module (from residues 24 to 618 approximately) featuring a first β-propeller fold composed of six blades (from residues 24 to 300 approximately), and a second β-propeller fold composed of seven blades (from residues 310 to 618 approximately). This type of organization has already been observed among carbohydrate processing enzymes, such as glycoside hydrolases from the clans GHE, GHF, GHJ, and for λ-carrageenases (Guibet et al., 2007), but also in the PL11 family of polysaccharide lyases involved in the degradation of anionic polysaccharides. The C-terminal module (comprising amino acids 663–1060 in *Psc* ι-CgsA) displays a typical TIM-barrel fold found in the amidohydrolase superfamily. This superfamily includes an outstanding set of enzymes that catalyze the hydrolysis of a wide range of substrate having amide or ester groups. For example, urease, amidohydrolase, guanine deaminases and phosphoesterase share the same three-dimensional fold describing this superfamily (Seibert and Raushel, 2005).

**Figure 6:**
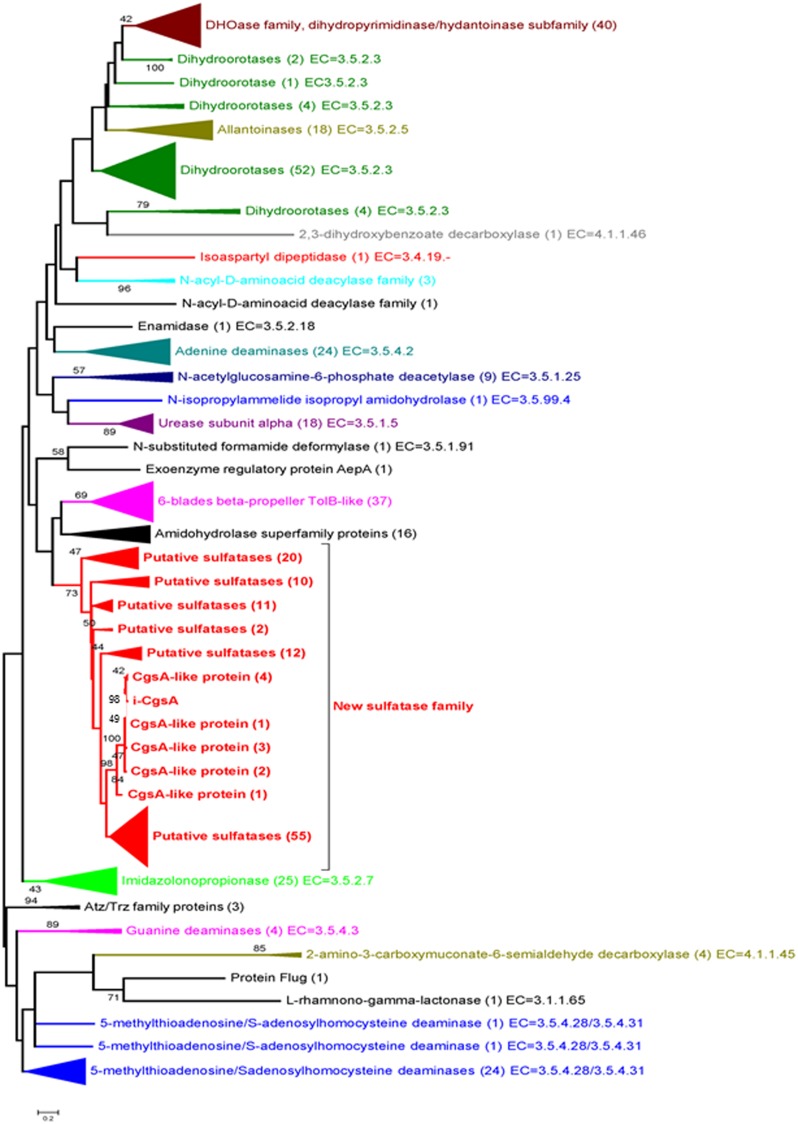
**Phylogenetic tree of the amidohydrolase module present in *Psc* ι-CgsA and in 420 sequences of enzymes classified as amidohydrolases**. The maximum likelihood tree was built with the MEGA v. 5.05. Software, using the substitution model WAG. Evolutionary rate differences among sites (5 categories) were modeled using a discrete Gamma distribution, from an alignment produced by the MAFFT program with the L-INSi algorithm (Katoh et al., 2005). Phylogenetic analysis was performed using 171 positions, defined from the multiple sequence alignment. All ambiguous positions were removed for each sequence pair. Only the bootstrap values higher than 40 are shown. The potential new sulfatase family (in red) was delimited on the basis of sequence similarities to the here identified sulfatase and the bootstrap value (73) of the best node (the deepest, the first red node) in the tree. Numbers in brackets indicate the number of sequences involved in each group.

A phylogenetic analysis of the C-terminal module found in *Psc* ι-CgsA and in 420 sequences of enzymes classified as belonging to the amidohydrolases superfamily showed that *Psc* ι-CgsA was not related to any known activity in this superfamily (Figure 6). In contrast, it appears that *Psc* ι-CgsA belongs to a clade composed of uncharacterized proteins only. In this cluster, a sub-clade supported by a node with a relatively good bootstrap value (73%) is composed of 122 sequences displaying the same modular organization as *Psc* ι-CgsA (i.e., the presence of β-propeller modules at the N-terminal extremity) (Supplementary Table 1). The topology of the phylogenetic tree suggests that *Psc* ι-CgsA and these 122 sequences represent a novel family of sulfatases, which might have different substrate specificities, as supported by the low percentage of sequence identity between some proteins within this clade.

### Production and analysis of the recombinant ι-carrageenan sulfatase

In addition to the identification of the native *Psc* ι-CgsA enzyme, the gene coding for *Psc* ι-CgsA was successfully cloned and heterologously expressed in *E. coli*, although the purified recombinant enzyme displayed lower activity on the natural substrate than the native form. The biochemical characteristics of the native and the recombinant enzymes, measured on the artificial substrate MUFS, are very similar (Table 3A), but the *kcat* of the native enzyme is roughly about 3 times that of the recombinant enzyme (Table 3B). The slightly different biochemical behavior of native vs. recombinant enzyme might be due to intrinsic elements in the native protein that *E. coli* does not supply, such as potential post-translational modifications and/or the requirement of a cofactor or a chaperone during protein folding, preventing the production of a fully active recombinant enzyme. Future work and a more detailed biochemical depiction of different recombinant homologs of this novel sulfatase family are necessary, and will shed more light on the precise catalytic mechanism and mode of action of this newly discovered enzyme family.

### Deciphering a new pathway for carrageenan biodegradation

The degradation pathway of κ-carrageenan by *P. carrageenovora* Psc^T^ has been partially determined and involves a κ-carrageenase (Weigl and Yaphe, 1966a; McLean and Williamson, 1979a; Barbeyron et al., 1994; Michel et al., 2001) to produce oligosaccharides which are readily degraded into neocarrabiose through the concerted action of a glycosulfatase (Weigl and Yaphe, 1966b; McLean and Williamson, 1979b) and a neocarratetraose monosulfate hydrolase (McLean and Williamson, 1981). For the catabolism of ι-carrageenan a similar pathway might be assumed as a low ι-carrageenase activity has been detected in the crude extract of *P. carrageenovora* Psc^T^ (Henares et al., 2010). However, based on our results with *Psc* ι-CgsA, we propose an alternative mechanism in which ι-carrageenan is first desulfated and converted into α-carrageenan. The latter likely constitutes a metabolic intermediate probably subject to further degradation by an α-carrageenase and/or desulfation by other sulfatases, leading to the end-product of galactose residues. Except for the conversion of ι- into α-carrageenan, these steps are speculative and need to be corroborated.

### Bioconversion of red algal polysaccharides

α-Carrageenan has been observed in the cell wall of several red algae such as *Catenella nipae* Zanardini (Zablackis and Santos, 1986), *Sarconema filiforme* (Sonder) Kylin (Chiovitti et al., 1998; Kumar et al., 2011) and in some *Solieria* spp. (Chiovitti et al., 1997; Bondu et al., 2010). Although structural studies revealed that this carrageenan referred to as hybrids of α-/ι-carrageenan in which the α-carrabiose content does not exceed 30–40% (mol/mol) (Falshaw et al., 1996), it has been shown that the sodium salt of such hybrids exhibit twice the capacity to suspend cacao particles in milk compared to κ-carrageenan which is commonly used for that purpose (Zablackis and Santos, 1986). As suggested by Figure 4, the Psc-ι-CgsA sulfatase could be used to produce carrageenan with controlled ratio of ι-/α-carrabiose and even pure α-carrageenan. It is therefore more than likely that new hybrids of α-/ι-carrageenans or pure α-carrageenan will harbor new and interesting functional properties.

Recent marine genomic projects have shown that marine bacteria are a potential source of large sulfatase diversity, with the presence of huge multigenic sulfatase families, as exemplified by the formylglycine-dependent sulfatase family (up to 300 genes in the *Lentisphaera araneosa* HTCC2155^T^ genome) (Thrash et al., 2010). Since the origin of these bacteria is marine, it is tempting to assume that a large portion of these enzymes are involved in the degradation of sulfated polysaccharides from marine algae (Glöckner et al., 2003). In addition to these sulfatases accessible through genome mining, our work demonstrates that the sulfatase diversity extends even beyond those that have already characterized members. By screening a marine bacterium for sulfatase activity on carrageenan substrate, we have successfully identified and characterized a new sulfatase family, members of which were annotated “hypothetical proteins with unknown function” before hand. Altogether, these data open up a large field for future work, necessitating substantial experimental effort to provide all the biochemical details to assign precise substrate specificities to these newly identified sulfatases. The immediate gain of such efforts will be a large panel of new marine polysaccharide sulfatases that represent interesting enzymatic tools to fine-tune the physico-chemical properties of algal polysaccharides.

### Conflict of interest statement

The authors declare that the research was conducted in the absence of any commercial or financial relationships that could be construed as a potential conflict of interest.
