# Supplementary material for: Discovery of a novel iota carrageenan sulfatase isolated from the marine bacterium Pseudoalteromonas carrageenovora
Source: Front Chem. 2014 Aug 26;2:67. doi: 10.3389/fchem.2014.00067 (PMC4144425; doi:10.3389/fchem.2014.00067)
Supplement: Supplementary file 1 [file DataSheet1.ZIP › 107201_Genicot_Supplementary_Figure_1.PDF]

Supplementary Material

Discovery of a novel iota carrageenan sulfatase isolated from the marine bacterium *Pseudoalteromonas carrageenovora*.

Sabine Genicot[1, 2*], Agnès Groisillier[1, 2], Hélène Rogniaux[3], Laurence Meslet-Cladière[1, 2, 4], Tristan Barbeyron,[1, 2], William Helbert[1, 2, 5]

[1] UMR 8227, Integrative Biology of Marine Models, Station Biologique de Roscoff, Sorbonne Universités, UPMC Univ Paris 06, Roscoff, France

[2] UMR 8227, Integrative Biology of Marine Models, Station Biologique de Roscoff, CNRS, Roscoff, France

[3] UR1268 Biopolymères Interactions Assemblages, INRA, Nantes, France.

[4] EA3882, LUBEM, Technopôle Brest-Iroise, Plouzané, France.

[5] Centre de Recherches sur les Macromolécules Végétales (CERMAV, UPR-CNRS 5301), affiliated with the Université Joseph Fourier (UJF) and member of the Institut de Chimie Moléculaire de Grenoble (ICMG, FR-CNRS 2607), Grenoble, France.

*** Correspondence:** Dr Sabine Genicot, UMR 8227, Integrative Biology of Marine Models, Station Biologique de Roscoff, Centre National de la Recherche Scientifique, Place Georges Teissier, Roscoff, 29680, France.
genicotl@sb-roscoff.fr

1. Supplementary Figures

Supplementary Figure 1. **Sequence alignment of *Psc* ι-CgsA and Q3IKL4_PSEHT from *P. haloplanktis* TAC 125 with 9 proteins chosen amongst the 120 ones representing the new sulfatase family has been performed using using the program MEGA 5.** The ESPript 3 program (Robert and Gouet, 2014) was used to enhance the conserved amino acids which are shown in white letters on a red background, whilst similar residues are written in red. The Genbank accession codes are given and are as follows: EHR39315: hypothetical protein AJE_17490 from *Alishewanella jeotgali* KCTC 22429; AAZ24146: amidohydrolase family protein from *Colwellia psychrerythraea* 34H; YP_154995: hypothetical protein IL0605 from *Idiomarina loihiensis* L2TR; ADY50984: amidohydrolase from *Pedobacter saltans* DSM 12145; ABV3463: amidohydrolase from *Shewanella sediminis* HAW-EB3; EAR27769: hypothetical protein PTD2_18145 from *Pseudoalteromonas tunicata* D2; EGD20757: amidohydrolase, imidazolonepropionase from *Xanthomonas gardneri* ATCC 19865; YP_002760548: hypothetical protein GAU_1036 from *Gemmatimonas aurantiaca* T-27; EAR01663: hypothetical protein FB2170_14083 from *Maribacter* sp. HTCC2170.

2. References

Robert, X. and Gouet, P. (2014) "Deciphering key features in protein structures with the new ENDscript server". *Nucl. Acids Res.* **42**(W1), W320-W324 - doi: 10.1093/nar/gku316 (freely accessible online).

```
                               1       10                  20        30
EHR39315       ................MLYFKPSLLALALACL..........PLQADTPAAEWQVNAPQG
AAZ24146       .........................MLALPTTFAKSPIDNEASIEETTP..KWSVNSPQG
YP_154995      ...................MKRTTLTLAVASLLSFAPVSM...AQEQTQ..GWDVSDPQG
ADY50984       .............MPMNTFFRIILFVLISSGLHA.................QWNIEKPSG
ABV34631       ................MLKHKLTPLCAAIALSFT.PPIFAEETLSEKEP..AWQVNAPAN
EAR27769       ................MKKFIYSSVAFAVAMTFS.PASFADEPKKEEKP..SWKVDEPQG
EGD20757       .............MKERGVRQWFGLGLMGCLSIAAAAALAHDPAEHLPL..PWLADAAGT
YP_002760548   MHSPPATPLFDELRVSSDRLAVGRLNPGGCLVLRSLPLSALALAVSLLPASSLVAQAAAG
EAR01663       ...................MKKLLLTLVL...FASISLFGQEDKKDTKTKEKWDISNPKG
Q3IKL4         ...........................................QQDDEP..KWQVDSPKG
Psci-CgsA      ...........................................QQDDEP..KWQVDSPKG

                                         40        50        60        70
EHR39315       ........E............FT..QVDIQVQQGSWMNVSVSPDGKTIVFDLLGDIYSMP
AAZ24146       ........N............FT..TADIDVRSGTWMNVDLNPDGKTIIFDLLGDIYTMP
YP_154995      ........E............FK..TIDISVNSGTWMNVDVSPDGEYIVFDLLGDIYRMP
ADY50984       ........P............TK..TTNFSTNEGTWMNLDVSPDGKFIVFDLLGDIYKMP
ABV34631       A.......P............LE..KVSIDVTEGTWMNVSVSPNGKHIVFDMLGDIYQIP
EAR27769       ........E............FF..DAKIAVEQGTWMNIDVSPDGKTIVFDLLGDIYTMP
EGD20757       AAAPSVPRPQQAPGQTLPLQATR..RIAFETDEGTWMGLDVSPRDGRLVFDLLGDLYTLD
YP_002760548   G.......ARGTPPRPLPLEATR..SYALDTREGTWLSVDISPDGKQIVFDMLGDLYTMP
EAR01663       ........Q............FNYIEHQFKTDEGTWMNLDVSPDGKTIVFDLLGDIYSIP
Q3IKL4         ........Q............FV..DASISVEQGTWMNVDISPDGETLVFDLLGDIYTMP
Psci-CgsA      ........Q............FV..DAAISVEQGTWMNIDVSPDGKTVVFDLLGDIYTMP

                     80        90       100       110       120        130
EHR39315       ISGGAATALTNDIAWNMQPVFSPDGKYIAFTSDRDGGDNIWIMRPDGSEPRAVT.KESFR
AAZ24146       ASGGEATPLMTDIAWQMQPRFSPDGKYIAFTSDEDGGDNLWIMKADGSEAKAVT.TETFR
YP_154995      FGGGEAEKLIGGIAWHMQPVYSPNGRYIAFTSDQGGGDNIWVMDADGNNVRAVT.EETFR
ADY50984       VTGGDAELLAGGLAWEVQPRFSPDGKLISYTSDKDGADNIWIMNADGSGKRAIT.KENIN
ABV34631       MKGGEAKVLAKGIAWQMQPVYSPDGKYIAFTSDEDGGDNIWVMEADGSNPRAVT.EETFR
EAR27769       FSGGKATQLTSDIAWQMQPRFSPDGKSIAFTSDADGGDNIWVMDVDGQNAKAVT.KETFR
EGD20757       ADGGRAKAISRGLGFDSQPTFSPDGRWIAFVSDRSGAENLWRMRPDGRDAQQLTFGDDDT
YP_002760548   FAGGEATRITNGLAFDAQPRFSPDGKSVVFISDRQGADNVHTIDLATNEIKEIT.RGRTN
EAR01663       ITGGNAKILRTGIPFEIQPRFSPNGKKIAFTSDAGGGDNIWTMNIDGSDAKQIT.KEKFR
Q3IKL4         MSGGKATKITSDIAWQMQPRFSPDGKHIAFTSDQGGGDNIWIMDVNGENQTAVT.DETFR
Psci-CgsA      MSGGTATKLTSDIAWQMQPRFSPNGKHIAFTSDQGGGDNIWIMDLNGENQHAVT.DETFR

                     140       150       160       170            180
EHR39315       LLNSPAWSPDGQFIVARKHFTASRSLGAGEVWQYHISGG.NGVMLTERP....NDQKDLG
AAZ24146       LLNSPAWSPDGNYIVGRKHYTGTRSLGAGEVWMYHKSGG.NGVMLTKRP....NEQKDLG
YP_154995      LLNSPAWSPDSEFLVARKHFTASRSLGAGEVWMYHRSGG.SGVQLTERP....NDQKDLG
ADY50984       LLNNAVWTPDGQYLVARKNFLGRRTNAAGELWMYHVSGGFEGTQLTQRK....RGQRNLG
ABV34631       LLNSPAWSPDSQYLVARKHFTGSRSLGAGEVWMYHVAGG.EGVKLTKRP....NEQKDLG
EAR27769       LLNSPAWSPDGDFIIARKHFTASRSLGAGEVWLYHKAGG.NGVQLTKRE....NDQKDLG
EGD20757       VLVSPAWAPDGSALYASR...FRWSVNDYELWRYGLDCS.ERLVAPVRAEGAGSEQSTLG
YP_002760548   VYLSPEYSPDGQYIVASK...GSFRGALPTLWMYHVRGG.SGVSLYTAP....ANAAP.G
EAR01663       LLNNVSWMPDGNYFVARKHFTSQRSLGAGELWQYHISGG.SGLQLTKRK....NDQQDVN
Q3IKL4         LLNSPAWSPDGDYLVARKHFTASRSLGAGEVWLYHKAGG.KGVQLTKRE....NDQKDLG
Psci-CgsA      LLNSPAWSPDGDYLVARKHFTASRSLGAGEVWLYHKAGG.KGVQLTKRE....NDQKDLG

                      190       200       210        220       230
EHR39315       E......PAFSPDGHYIYFSQDDTPGKTFHYSQDSES.GIYAIKRFERATGNIEVLLAGA
AAZ24146       E......PAFSHDGKYVYFSQDATPGKSFHYSKDSEK.GIYKIKRLELETGEIKVVVSGK
YP_154995      E......PAFSPDGKYIYFSQDDTPGKTFHYSKDSLE.GIYDIKRFERETGEIETLISGA
ADY50984       E......PFVSKSGKELYYSEDISPA.YLQYNKDPNG.EIYQIKRLILEDETVENVAGGT
ABV34631       E......PAYSPDGRYIYFSQDATPGKTFHYSKDSVK.GIYKIKRYDTQTGDIEVLIEGT
EAR27769       E......PAFSPDGRYVYFSHDATPGKTFHYSKDSVD.GIYKIKRYDRQTGEIETIISGM
EGD20757       .......AVVSPDGRQLYAARRSG.......DKDSAELELWSIVRRDLATGKEETILPVP
YP_002760548   TAVQQAGASFSPDGRYLWYTQRTG...AWHYNAQFPQ...YQVWTYDRETGEREIQSTRY
EAR01663       E......PNISPDGKYMYYSEDVYPGGYFQYNKDPNK.QIYVIKRYDFETGKTIIVTGGP
Q3IKL4         E......PMFSPDGRYVYFSHDATPGKTFHYSKDSVA.GIYKIKRYDRETGDIETIISGM
Psci-CgsA      E......PMFSPDGRYVYFSHDATPGKTFHYSKDSVA.GIYKIKRYDRETGEIETVIDTM

             240                250       260       270       280       290
EHR39315       GGAIR.........PTPSPDGKYLAYIRRVDFQSTLFLYDLTSGEHIKLYDGLDRDMQET
AAZ24146       GGAIR.........PTLSPDGKYLAFISRDDFQSNLYLYNLKNGEQSKVYDGLDRDMQET
YP_154995      GGAIR.........PTPSPDGRYLAYIKREDFDSVLYVLDLKTGEHKRVYGKMERDMQET
ADY50984       GGAVR.........PQVSPNGKYLAYVKRNKLKTNLMIQDLETGEEFILYDDLSRDLQES
ABV34631       GGAIR.........PTPSPDGKKLAYIKRDGFQSSLYMLDLRSGETEKLYGKLDRDMQET
EAR27769       GGAIR.........PTPSPDGKTLAYIKRDDFQSSLYLYDLATGQHTKVYGAMERDMQET
EGD20757       GVPGRRPFPGTYFSPRLSPDGKQLAYATRQQGQTGLRLRTLATGEDRWIAFPIAHDQSQA
YP_002760548   GSAVR.........PTVSPDGKWMVYGSRYENKTGLRIRDLASGDERWLAYPTQRDEMET
EAR01663       GGAAR.........PQVSRDGKKLAFIKRVRTKTVLFIHDLETGEEWPVIDSLNKDQQEA
Q3IKL4         GGAIR.........PTPSPDGKKLAYIKRDDFQTSLYLYDLTSGEHTKLYDKLERDMQET
Psci-CgsA      GGAIR.........PTPSPDGKKLAYIKRDDFQTSLYLYDLSSGEHTKLYDKLERDMQET
```

```
                       300       310       320       330       340       350
EHR39315        WAIHGVYPTLSWTPDNQQLVFWAGGEIKTLRLSDKSVSTIPFSVNTSKQIQTALRVKQDL
AAZ24146        WAIHGVYPTMAWTPDSEGLVFWSGGKINKLSLDSKKAKVIDFHVKTTKKIQTAVRFPQDL
YP_154995       WAIHGVYPTMDWTPDSKQLVFWAGGHIQKLNIDDGSARIIPFKVETEKKVQHALGFKQNV
ADY50984        KASYGLYPNFNWLPDNKTVIFYAKGKINKIDILYQMVQEIPFKVNVKQTISEALHYDQKV
ABV34631        WAIHGVYPTMSWTGDNEEIVFWAGGKINKLDVESKSVKQIPFSIKTQLDVQPAVRFTQDL
EAR27769        WAIHGVYPTMAWTPDNKGLVFWAGGKIHQVDVKTKQASEIAFKVETSKKMQKALRFAQNI
EGD20757        QSWQDLVPRYAFSRDGRALLLSRNGRFERIALDREAPRAIPFIASVDLELGPLTGADIRQ
YP_002760548    RAPLDALPGMSFTPDSKEIVASYGKKLWRVAVDGSGQVEIPFHVKADVAVGPEVSFTYPI
EAR01663        WAIFGVYPSFSWLPNDKGIVFWNKGKIHKVDINTLVVTNIPFTVDAKIKIAETVHFDSPV
Q3IKL4          WAIHGVYPTIAWTPDNEELVFWAGGTLHKFNVDNKSVSDIAFKVDTTKKIQKAVRFTQNI
Psci-CgsA       WAIHGVYPTIAWTPDNEELVFWAGGTIHKLDVADKSVKTIPFKVQTSKKIQKAVRFTQNL
```

```
                        360       370       380       390            400
EHR39315        .DQSQFDTKMLRFVQVSPDGKQAVFTALGHLYITELNNPR.PR....RLTR.QTTDFEFY
AAZ24146        .DQKSFDTKMLRDVKVSPNGKLAVYESMGHLYTKSLPKGK.PK....RLTK.QKSQFELN
YP_154995       .EADQFDVKMLRMAQVSPQGDAVIYEALGSLYQRSLPDGE.PQ....RLT..NSEDFELY
ADY50984        .FNEEFDAKMIRQLVTSPDLKKVAFSAGGYIYVKNLPDGK.PE....RLT..TETDWEFD
ABV34631        .DKDKFDVKMLRMAQVSPDGKKVVYEALGKLWVKSLSDGK.SDGKRTRLTKLDSELRELF
EAR27769        .DTPEFDVKMLRNVQISPDGETAIFEAMGKIYLRDLDSGK.IK....PLTK.SSAGFQFF
EGD20757        .DTGPVQARLIQDPAVSPDGTRVAFSALGALYLMDMEDGALPK....RLP..TGDVPAFH
YP_002760548    SDSAQFTVRQIRDGVMSPDGKQLAFISLDKLYVMDWPSGT.PR....RVS..TLNDIEAE
EAR01663        .APEEFSAKVIRHAVTSPDKKTLVFSALGHLWTKRLPNGK.PK....RLT..KSENLEFE
Q3IKL4          .DTDEFDVKMLRNVQISPDGETALFEALGYIYMRDLESGK.IK....RLTK.QTDHYELF
Psci-CgsA       .DTDEFDVKMLRNVQVSPDGATAIFEALGYIYKRDLESGK.IK....RLTK.QTEHYELF
```

```
                    410         420       430       440        450       460
EHR39315        PQFSRDGRELVYVSW..NDQTQGAVNILNLRNNRVTKLTTEPGK.FIEPTFSPDGRTVVY
AAZ24146        PSFSRDGKKIVYVSW..DDKNLGQVRVVSSRGGKGKTITREPGK.YVEPSFSPDGKAVVY
YP_154995       PQYSRDGEKVVFVRW..NDEAQAKVIVRDISSGDEQVLNTGQGN.FVEPTFSPNGKHVVY
ADY50984        PCFSPDGNFIIYTSW..NDELKGSIMKIDLSSKKIDKLTPRGGF.YYTPKFSNKGDLIVY
ABV34631        PQWSRDGKSIVFTTW..DDLEQGSVRVVSLRNKRVKTLTKEPGK.YVEPTFSPDGSFVVY
EAR27769        PTFSRDGKQVAFVSW..QDDEQSQLHVVSTRSGKGKTLLKEKGK.FVEPVFSPDGKTVVF
EGD20757        PSWAPDGKALTYVTW..EGGEGGQVWALAPGAAAPRQLSHGPGAYYTHPVFTPDGRDVLV
YP_002760548    PAWSPDGRSLAWVTWANNGGRLYKSAMVAGRATRVLPVSASTGT.FRQPVWSPNGARILV
EAR01663        PSFSADGKKLVYVSW..NDESLGAIHSIPVTGGTPSKLTTQKGI.YRTPTYSPDGKMIVY
Q3IKL4          PQYSRDGKNIVYTTW..DDNEQGTVRVVSARSGRGDTITQEPGK.YVEPTFSPDGKTVVY
Psci-CgsA       PQYSRDGKKIVYTTW..NDNKQGTVRVVSSRSGRGDTITTEPGK.YVEPTFSPNGKTVVY
```

```
                             470       480        490        500       510
EHR39315        ......RKIGAGSLLPKQWSMNTGLYKVSSRGG.EAELIARN..GRAPMFGAGNDKVYAM
AAZ24146        ......RKVTGGFITDPTWGLNPGVYAVSVKGG.KAKLVTEE..GVQPHFGARNDRIYVT
YP_154995       ......RKVRGGYLISDKFGLENGIYRVAVSANAEPEQIAES..GHLPQYGARNDRVYLM
ADY50984        ......RKGVGNDLLGFTFSKNPGIYTISANGG.EPKLILKE..GLKPQFNKSDNRIFYT
ABV34631        ......RKAKGGYITPRSWSQETGLYRVDTKGK.ENIKITPD..GYQPQFGAESDRVYFM
EAR27769        ......RKVRGGAILDPDFSLNPGIYKVPSTGG.DAELIFKQ..GEQPQFGKDNDKIYVL
EGD20757        VASDNVARMQASMVFGSV..REAQLQRLSAQGG.QARILHRGTLGGTLHFGADADRVYAM
YP_002760548    LQSGAQGRRDQTGVTGTTQLMWFAANSTSTNGD.AGTLIARAAGRSKPHFSRDTTRIYLS
EAR01663        ......GKESGNNDQGRTFSKKSGLYTMSANGT.NPKWISKE..GEYPQFTANGKRIFYR
Q3IKL4          ......RKASGGNILNPKWSLHPGVYSVSAKGG.KSELISKS..GYQPQFGSANDRVYIM
Psci-CgsA       ......RKATGGSILNPKWSLNPGIYSVSVKGG.KSELISKS..GYQPQFGDESDRVFIM
```

```
                           520       530
EHR39315        DFGS.....APRLISIDLTKHTQQTLYSAK..............................
AAZ24146        RDGE.....TPHISRIDIDGQHDTKLYQGK..............................
YP_154995       TPGD.....KPTLSAIDLRTLEQRDLYQSE..............................
ADY50984        AKED....GKNALKSCDLSGNNIRTHYTTK..............................
ABV34631        DHGE.....TPELASINLDGFQKRVHYTSK..............................
EAR27769        GIGE.....KPSLSMIDLNTKKAQKLYESE..............................
EGD20757        GSGG........LLGIDLRSGLADAPIVVKGPGWYFQDGSV...................
YP_002760548    SNNG........LVSIRWDGTDEQRHLRVVGGG...GDGAADDDHAHTDMTVAELELQRT
EAR01663        TGGTYFGNLTKTLHSIDLNGKDKRNHIKSK..............................
Q3IKL4          SPWP.....KPTLSVVELESKKVRKLYESE..............................
Psci-CgsA       SPWP.....KPTLSVVDLKSKKIRKLYESE..............................
```

```
                          540       550       560         570        580
EHR39315        .........FGTEFKLSPDGKYLAFADRFKVFVTPFVERGR..PIEI.AANDKQFPVKQL
AAZ24146        .........FASEYRVSPDGNYLAFAERFKVFVTPFVERGD..VIDI.GPKAENLPVEQL
YP_154995       .........LATEFRVSPDGKQLAFAERFKVFVTPLVERGS..VINV.GPGDKQMPVHQL
ADY50984        .........LGTEFILSPDNRWLAFSELFDIYLTPFIATAN..GTEL.ASNTKSFPVKKV
ABV34631        .........HATEFRVSPDGKQLAFAERFKVWVTPFAKHGE..TIEI.GPKANNLPVTQL
EAR27769        .........LATEFRISPDSRYLAFAERFKVFITPFVASGK..TINI.GPKDTQFPLEQL
EGD20757        .........PVDDLRISPDGQWLLAQVAQQLHLLPVPKKGT..TVDLLAPGMRHRRITDV
YP_002760548    PEEPNSPGPPAQLVMISPNGDVALAQINQDFYTVTIPPRGTQATVNIADPNTASFPARKL
EAR01663        .........YANLLVPSPDNKWVAFTNLHKAFIAPLNLNGK..TVDL.DNNSKSVPVSQI
Q3IKL4          .........HATEFRVSPDGEYLAFAERFKVLVTPFVERGK..TINI.GPKDSQFPIEQL
Psci-CgsA       .........HATEFRVSPDGQYLAFAERFKVFVTPFVESGS..TINI.SPKDSQFPIEQL
```

```
                  590       600       610       620
EHR39315      SVRAGEYISWAGDSSSLYWSLGPELYQQQVSGLFDLSA.................AKSPE
AAZ24146      SMRAGEGINWNGKSNELYWSLGADLYQASIKGLFDITAKTTDENDDGSEPETSASTVVND
YP_154995     SVRAGENLSWTANSDQLYWTLGPELYHTSVAEAFNADTES...............DTLKV
ADY50984      SGDVGNYIHWNKESKALHWTLGSRYYTKEIKDALAFSNDT...............KDKSD
ABV34631      SVRAGESISWNNDSDQLYWTLGPELYQVEVDTAYAAKDKTADDKQ..........AKAIE
EAR27769      SVRAGENISWNTDADKLYWTLGPELYHADLNGIFDIKSAK...............NDFKV
EGD20757      G...ADYFAWSADGRTMTWSIGSSVYRRARADIALHVADAPGWDADVP.......TPGRN
YP_002760548  TDIGGQFPAWSSDGKRLHWSIGNAFVSFDVDSAAARDAAIAIARRDSVPEASR..PRPYV
EAR01663      AKDAGINLHWSKDSKTIFWTLGDEYFSNNIKDRYTFLPGSPEK............VTAMD
Q3IKL4        SVRAGENISWSANSNKLYWTLGPELYHASLEGMFAINKADD..............KDVKV
Psci-CgsA     SARAGENISWNTKSNTLYWTLGPELYHASLEGMFAINKADD..............ADFKV

              630       640       650       660       670       680
EHR39315      PKITQLGFSAPVDIPRGSVAFVGGQVITMQGDKVLQDAVVLVRDNKISAVGSRDEIAIPK
AAZ24146      INVINLSYKQKVDIPSGHVAFVGGKVITMEGEQVIDNGVVLVEGNKIKAVGTKGQVNIPS
YP_154995     ENGQNIGFEHDAMIPDTTVAFVGGQVITMNGDTVYKQGTVVVRNNKIVAVGPEAEVNVPS
ADY50984      TLGLEIALTLKTDVPQHKIAFKGATIITMKDNEIIPNGTILIDQNKIVAIGKDNEVNIPD
ABV34631      PQITDIGFSQNVDVPRGTVAFVGGKVITMEDDKVIENGIVIVKNNHIVAVGG.ADTEVPD
EAR27769      ADGANISFKQTMAEPNGLVALTGARIITMNGDNVIENGVVLTDGKHIKAVGAVGAVSIPS
EGD20757      TQMVQVRVDVPRDVAHGTLLLRGATALTQRGDEAIADADLLVRDGRIAAIGPRGSVEVPA
YP_002760548  PLEQRIVMQGTRDIPSATVVLRNAKLVTMKGDEVIARGDVVVKNNRITAIGAAGTVTVPS
EAR01663      SVGLKIGLTGKTDRPEGRIAFTNARIITMEGDEVIENGTILIHENRIEKLGNSGDIKIPS
Q3IKL4        KSGTNIGFSKKMAEPQGMIALTGAKIITMEGEKVIENGVIVTDGKHIKAIGTAADVSIPK
Psci-CgsA     KSGDNIGFSKKMAKPKGMIALKGAKIITMDGEKVIENGVIITDGKHIKSIGTANEVTIPK

              690       700       710       720       730       740
EHR39315      DARVFDISGKTLMPGLFDAHAHGSQGVQQITPQQNWQNYAALTFGVTSIHDPSNDTREIF
AAZ24146      DATIIDITGKTVMPGLIDAHAHGPQGSNEIIPQQNWKNYAGLALGVTTIHDPSNDTTEFF
YP_154995     GAEVIDTSGKTVMPGLFDAHAHGSQGQAEIIPQQNWGQYANLAFGVTSIHDPSNDTTEFF
ADY50984      DAIVYPAYGKTIIPGFIDVHAHLKTSPDGIIPQQNWAYYANLAYGITTAHDPSTNMEMAS
ABV34631      DAQVIDIKGKTIMPGLFDAHAHGSQGEDEIIPQQNWELYSNLSLGVTTIHDPSNDTTEIF
EAR27769      NAKVVDVTGKTIMPGIVDAHAHGAQGSNEIIPEQNWKNLAGLALGVTTIHDPSNDTSEIF
EGD20757      GAQLRDVSGRFIIPGLIDVHDHVADFRRDLLDMRPWGLRARLAYGITTAFDPSSLSIDML
YP_002760548  GATEMDLAGATIIPGFVDTHAHLRAERGSIHESQPWAYLANLAFGVTTTRDPQTATTDVL
EAR01663      GVKIYDVEGKTIMPGIVDAHAHIGGFRYGLATQKHWQLYANLAFGVTTVHDPSANTESIF
Q3IKL4        GAKVIDVTGKTIMPGIVDAHAHGSQASDEIIPQQNWKNLAGLALGVTTIHDPSNDTTEIF
Psci-CgsA     DAKVIDVTGKTIMPGIVDAHAHGSQASDEIIPQQNWKNFAGLALGVTTIHDPSNDTTEIF

              750       760       770       780       790        800
EHR39315      SASELQRSGQIVGPRIFSTGTILYGAEGAGYTSHIDSLDDARFHLTRLQK.VGAFSVKSY
AAZ24146      AASEMQKAGKIVAARLFSTGTILYGATIPGYTSHVDSLDDAKFHVERLKA.AGAFSVKSY
YP_154995     AASELQKAGRIAAPRLFSTGTILYGANGPGYTSHVDSLEDAKFHLERLKK.VGAFSVKSY
ADY50984      SQAEMIKAGRLVGPRLFAAGSIT.GDKDTEIKTGFQNLAEIKSHLKRMQA.IGTFTLKSS
ABV34631      AASEQQKAGNIAGPRIFSTGTILYGANGPGYTSHIDSLDDAKFHLERLKK.VGAFSVKSY
EAR27769      AASEMQKAGKIVGPRIFSTGTILYGANGPGYTSHVDSLDDAKFHLERLQK.VGAFSVKSY
EGD20757      AYQDMVDAGMVVGARAPTTGMAMFSFN......RIASLDEARALLRRYRDHYRTRTVKQY
YP_002760548  TYQDLVETGQIIGPRVYSTGPGIFSTD......NIRDQEHARSLLKRYSSYYDTKTIKMY
EAR01663      TMSEMVKNGTMVGPRIYSTGFILYGADG.DFKAVVNNLEDAKSAIRRTKA.FGAKSVKSY
Q3IKL4        TASEMQKAGMIVGPRIFSTGTILYGANMPGYTSHIDSLDDAKFHLERLKK.VGAFSVKSY
Psci-CgsA     TASEMQKAGMIVGPRIFSTGTILYGANMPGYTSHIDSLEDAKFHLERLKK.VGAFSVKSY

                810       820       830       840       850       860
EHR39315      NQPRRNQRQQVIQAARELNMMVVPEGGSLMQHNLTMIVDGHTTIEHALPAARVYQDIKQL
AAZ24146      NQPRRNQRQQFIQAARELEMMVVPEGGSLLQHNLTMVVDGHTTLEHSISTAKIYADIKQL
YP_154995     NQPRRNQRQQVVEAGRELEMMVVPEGGSLLQHNLTMIMDGHTTVEHSLPVANIYDDIRQL
ADY50984      GLYRRDQRQQLVQAAREQKMMIIPEGEYDFFTSMNMIADGHSSIEHNIPVLPVYKDVSQF
ABV34631      NQPRRNQRQQVIAAARELEMMVVPEGGSLLQHNLTMIADGHTGIEHSLPAASIYNDIKQF
EAR27769      NQPRRNQRQQIIEAGRELGMMVVPEGGSLLQHNLTMVVDGHTGIEHSIPPEHIYDDIKQL
EGD20757      LVGNRRQRQWLVQAAHELGMRPTSEGSLALKLDLNQVMDGYAGHEHALPT.PLYRDVIEL
YP_002760548  VSGVRQVRQWIITAAREQQLMPTTEGSLDVKLNLTETLDGYPGLEHSIGIVPLGADIVNF
EAR01663      NQPRREQRQQILQAARELGINVVPEGGSTFYANMTMVMDGHTGIEHNIPVAPVYKDVIEL
Q3IKL4        NQPRREQRQQVIEAGRMLEMMVVPEGGSLLQHNLSMVVDGHTGIEHSIPVEHIYDDIKQL
Psci-CgsA     NQPRREQRQQVIEAGRELEMMVVPEGGSLLQHNLSMVVDGHTGIEHSIPVEHIYDDIKQL

                870       880       890       900       910       920
EHR39315      WQASGTAYTPTLNVAYGGIWGENYWYDKTEVWKHPRLSRYVPMDQLAPRSMRRP......
AAZ24146      WSQSDMAYTPTMGVAYGGISGENYWYDTTNVWEHPRLSQYVPSEFLDPRSMRRP......
YP_154995     WQQSDVAYTPTLGVAYGGIWGENYWYAETDVWKHPKLTQYVPMEFLVGKAMRRE......
ADY50984      WNASKTSYTPTLIIANGSQFGENYWYDRINVWENQKLLNFIPRHLVDSKSRRRT......
ABV34631      WSQTKVGYTPTLVVAYGGISGENYWYDKTDVWAHPRLSMYVPGDLLQARSMRRP......
EAR27769      WSQTEVGYTPTLGVAYGGIWGENYWYDKTDVWKHPRLSKFVPKNQLFPRSMRRT......
EGD20757      MARSGTSYDATLMIANGGPAAQNNYVIRDRPLGDATFRVTRPYEVAMQQVQSQR......
YP_002760548  LAWSKRTYTPTLLVNYGGPWAENYFYTKEDPWANPKLQRFTAYEELALKTRRRMASMPNG
EAR01663      WKTSGSGYTPTLIVNYAGMSGEYYFYQKDNVWENEKLLKYTPRAIIDARSRHRV......
Q3IKL4        WSQSDVGYTPTLVVAYGGIWGENYWYDKTDVWNHPRLSKFVPKNQLLPRSMRRV......
Psci-CgsA     WSQSDVGYTPTLVVAYGGIWGENYWYDKTDVWNHPRLSKFVPKNQLLPRSMRRV......
```

```
                            930       940       950       960       970
EHR39315      .....TAPEHHY..NHFNVVKTANELKEVGVVANIGAHGQREGLGAHWEIWMFAQGGMTA
AAZ24146      .....KAPLHHY..NHFNVAKVAKEMQDLGVVVNAGGHGQREGLAMHWEMWMMAQGGMSP
YP_154995     .....KAPHNHY..NHFNNAEVATELQDLGVEVLAGAHGQREGLAQHWEIWMMAQGGMEP
ADY50984      .....TSEYGDY..GHIDISRVVNQIANGGTKVNLGSNGRLQGLGAHWELWMLGQGGMKP
ABV34631      .....TAPDEHY..NHFNVARVANEMNDLGVKPNIGAHGQREGLAAHWEMWMFAQGGMSN
EAR27769      .....KAPDHHY..NHFNAAKVAAELQNVGVKVNLGAHGQREGLAAHWEIWMFAQGGMTP
EGD20757      .....WIDPSLM..LYPRIAGDVARIQRAGGLVAMGSHGEIAGPGLHWEMQAHVEGGMTP
YP_002760548  GTAGGWFRDDEY..IFPQLATDAAGVLRAGGRIGVGSHGQLQGLGYHWELWAMATGGLTT
EAR01663      .....MVPDEEYENGHILTSKTVTDLSQAGVKVNLGAHGQLQGLGAHWELWMLHQGGMTN
Q3IKL4        .....KAPDHHY..NHFNNARVAAELQDLGVLVNLGAHGQREGLGAHWEMWMFAQGGMTS
Psci-CgsA     .....KAPDHHY..NHFNNARVAKELQDLGVLVNLGAHGQREGLGAHWEMWMFAQGGMSS

                  980       990      1000      1010      1020      1030
EHR39315      LEALRTATLNPAITFGMDHQLGSIEVGKLADLIVINGDPLQDIRVSDQVIYTMVNGRLFD
AAZ24146      LQAIRTATISPAKSLGLDKDLGSLTVGKLADMIVIDGDITQDIRLSDKVTHTMINGRLYN
YP_154995     LEALRTATIDPARVFGMDHALGSLQEGKLADIIVIDGNPLENIRDTDKVTHTMVNGVLYD
ADY50984      HDILKVATINGAEYLGMDRELGSLEKGKLADLIVLNRDPLQDIRNTESIKYVMVNGRLYD
ABV34631      LEVLKTATINPAKHFAMDHQIGSIEQGKLADLIVIDGDPLEDIRITDRVTYTMVNGKLYN
EAR27769      LEALRAATIDPAHYLGLDKHIGSIEAGKLADLIVIDGNPLEDIRVTDKVTYTMINGQLFD
EGD20757      VEVLQAATLGGARSIGRGAELGSLEAGKLADLVILQADPRLDIRNAQKIDAVMLGGRLRK
YP_002760548  LEALRVSTIIGATAIGLQNDLGSLEVGKLADLLVLDRDPTTDIRNTNSIRYVMKNGRLYD
EAR01663      HEALQTATINGANYIGAGKEIGSLKEGKLADLIVLENNPLEDIRNTESVIYTMANGRLYD
Q3IKL4        LEAIRASTLDPAKYLGLDKNVGSLEVGKLADLMVIDGDPLKNIRDSDKIDYTMINGRLFD
Psci-CgsA     LEAIRASTLDPAKYLGLDKNVGSLEVGKLADLMVIDGNPLKNIRDSDKVDYTMINGRLFD

                 1040          1050
EHR39315      AETMHEIG....LRERQRQPFYFEAR....
AAZ24146      ADTMNEIG....NYDNKREKFYFENKGTGN
YP_154995     ATTMDRLL....PEPLKRKDFFWEE.....
ADY50984      AESMNELG....QDIKPINKFWWQFNNSEN
ABV34631      AETMNQLNGGSKNRGKERKPFFFENRTND.
EAR27769      AATMQAVD......GKTREKLYFEQ.....
EGD20757      VPTLDELW....PRESRIPALWHHGDALH.
YP_002760548  GNTLAE......QYPTKRTGPEVPNRPITP
EAR01663      TDTMHEIG....NNTNNRGMFWFENNKYN.
Q3IKL4        AATMNEVG......KKQRKPLYFENNK...
Psci-CgsA     ASTMNEVG......KKQRKPLYFENNK...
```
