# Supplementary material for: Discovery of a novel iota carrageenan sulfatase isolated from the marine bacterium Pseudoalteromonas carrageenovora
Source: Front Chem. 2014 Aug 26;2:67. doi: 10.3389/fchem.2014.00067 (PMC4144425; doi:10.3389/fchem.2014.00067)
Supplement: Supplementary file 1 [file DataSheet1.ZIP › 107201_Genicot_Supplementary_Table_1.PDF]

Supplementary Material

Discovery of a novel iota carrageenan sulfatase isolated from the marine bacterium *Pseudoalteromonas carrageenovora*.

Sabine Genicot[1, 2*], Agnès Groisillier[1, 2], Hélène Rogniaux[3], Laurence Meslet-Cladière[1, 2, 4], Tristan Barbeyron, [1, 2], William Helbert[1, 2, 5]

[1] UMR 8227, Integrative Biology of Marine Models, Station Biologique de Roscoff, Sorbonne Universités, UPMC Univ Paris 06, Roscoff, France

[2] UMR 8227, Integrative Biology of Marine Models, Station Biologique de Roscoff, CNRS, Roscoff, France

[3] UR1268 Biopolymères Interactions Assemblages, INRA, Nantes, France.

[4] EA3882, LUBEM, Technopôle Brest-Iroise, Plouzané, France.

[5] Centre de Recherches sur les Macromolécules Végétales (CERMAV, UPR-CNRS 5301), affiliated with the Université Joseph Fourier (UJF) and member of the Institut de Chimie Moléculaire de Grenoble (ICMG, FR-CNRS 2607), Grenoble, France.

*** Correspondence:** Dr Sabine Genicot, UMR 8227, Integrative Biology of Marine Models, Station Biologique de Roscoff, Centre National de la Recherche Scientifique, Place Georges Teissier, Roscoff, 29680, France.

genicot@sb-roscoff.fr

1. Supplementary Figures and Tables

1.1. Suplementary Tables

Supplementary Table 1.

Table S1. List of the 122 sequences belonging to the potential new sulfatase family

Table S1. List of the 122 sequences belonging to the potential new sulfatase family

Accession numbers	Descriptions	Organisms	Locus/Genes	Amidohydrolase module. From - to
A3HV30	Amidohydrolase family protein	*Algoriphagus* sp. PR1	ALPR1_02135	790 - 1177
J1QE69	Uncharacterized protein	*Alishewanella aestuarii* B11[T]	AEST_33180	644 - 1032
I8UAE6	Uncharacterized protein	*Alishewanella agri* BL06[T]	AGRI_02188	644 - 1032
H3ZJD0	Uncharacterized protein	*Alishewanella jeotgali* Bae MS1[T]	AJE_17490	644 - 1032
A0Y053	Putative uncharacterized protein	*Alteromonadales* bacterium TW-7	ATW7_06673	653 - 1041
F2G721	Amidohydrolase	*Alteromonas macleodii* Deep ecotype	MADE_1017735	673 - 1065
F5ZFR2	Amidohydrolase	*Alteromonas* sp. SN2	ambt_19300	673 - 1065
F4QZ84	MdsD protein	*Brevundimonas diminuta* ATCC 11568[T]	BDIM_07750	661 - 1048
B8GXK6	TolB protein	*Caulobacter crescentus* NA1000	CCNA_00115	684 - 1074
Q9ABV4	Putative uncharacterized protein	*Caulobacter crescentus* CB15[T]	CC_0116	665 - 1055
D5VDT9	Amidohydrolase	*Caulobacter segnis* TK0059[T]	Cseg_0112	680 - 1070
Q481I1	Amidohydrolase family protein	*Colwellia psychrerythraea* 34H	CPS_2573	660 - 1048
A8NHI7	Amidohydrolase	*Coprinopsis cinerea* Okayama-7#130	CC1G_10835	708 - 1115
H8MTH3	WD-40-like repeat-containing amidohydrolase	*Corallococcus coralloides* M2[T]	tolB2	717 - 1105
G0J5B5	Amidohydrolase	*Cyclobacterium marinum* Raj[T]	Cycma_3840	654 - 1043
Q2N7K8	Putative uncharacterized protein	*Erythrobacter litoralis* HTCC2594	ELI_11205	677 - 1066
A3WGV2	Putative uncharacterized protein	*Erythrobacter* sp. NAP1	NAP1_14738	661 - 1054
A5PE93	Putative uncharacterized protein	*Erythrobacter* sp. SD-21	ED21_22118	702 - 1091
E1SUL8	Amidohydrolase	*Ferrimonas balearica* PAT[T]	Fbal_0009	651 - 1039
C0BMP9	Amidohydrolase	*Flavobacteria* bacterium MS024-3C	Flav3CDRAFT_0650	751 - 1135
A8UL11	WD40-like repeat/amidohydrolase domain protein	*Flavobacteriales* bacterium ALC-1	FBALC1_06633	713 - 1097
H3NSB0	Amidohydrolase, imidazolonepropionase	Gamma proteobacterium HIMB55	OMB55_00007460	681 - 1069
F3L3V8	Amidohydrolase family protein	Gamma proteobacterium IMCC3088	IMCC3088_2300	650 - 1038
C1A768	Uncharacterized protein	*Gemmatimonas aurantiaca* T-27[T]	GAU_1036	741 - 1135
C1A9S7	Uncharacterized protein	*Gemmatimonas aurantiaca* T-27[T]	GAU_2212	700 - 1083
C1AAB0	Uncharacterized protein	*Gemmatimonas aurantiaca* T-27[T]	GAU_2666	749 - 1141
H2BWD9	Amidohydrolase	*Gillisia limnaea* FR 136[T]	Gilli_1211	665 - 1054
G4QMN6	Amidohydrolase	*Glaciecola nitratireducens* FR1064[T]	GNIT_2891	673 - 1064
H5TEI4	Putative uncharacterized protein	*Glaciecola punicea* ICO67[T]	GPUN_2596	688 - 1080
F4AL34	Amidohydrolase	*Glaciecola* sp. 4H-3-7+YE-5	Glaag_0537	676 - 1068
A0LY49	WD40-like repeat containing amidohydrolase family protein	*Gramella forsetii* KT0803[T]	GFO_0308	663 - 1052
D0LK65	Amidohydrolase	*Haliangium ochraceum* SMP-2[T]	Hoch_0458	704 - 1091
F4L3B0	Amidohydrolase	*Haliscomenobacter hydrossis* O[T]	Halhy_3894	746 - 1130
F4L562	Amidohydrolase	*Haliscomenobacter hydrossis* O[T]	Halhy_0879	642 - 1031
C6XP21	Amidohydrolase	*Hirschia baltica* DSM 5838[T]	Hbal_2526	661 - 1066
Q0BZS0	Amidohydrolase family protein	*Hyphomonas neptunium* 14-15[T]	HNE_2328	676 - 1063
Q5QWZ5	Uncharacterized conserved secreted protein	*Idiomarina loihiensis* L2-TR[T]	IL0605	648 - 1036
F7RYM4	Amidohydrolase, imidazolonepropionase	*Idiomarina* sp. A28L	A28LD_1357	649 - 1037
I0WH44	Amidohydrolase	*Imtechella halotolerans* K1[T]	W5A_05828	718 - 1102
C7R9R9	WD40 domain protein beta propeller	*Kangiella koreensis* SW-125[T]	Kkor_2530	660 - 1047
A9E6G3	WD40-like repeat/amidohydrolase domain protein	*Kordia algicida* OT-1[T]	KAOT1_01799	657 - 1044
B0D3C9	Predicted protein	*Laccaria bicolor* S238N-H82	LACBIDRAFT_315857	868 - 1264
A4AQC4	Putative uncharacterized protein	*Maribacter* sp. HTCC2170	FB2170_14083	659 - 1048
Q0AP15	Amidohydrolase	*Maricaulis maris* MCS10	Mmar10_1680	653 - 1041
A0Z4E9	Putative uncharacterized protein	Marine gamma proteobacterium HTCC2080	MGP2080_10073	662 - 1054
B7RTU7	Amidohydrolase family protein	Marine gamma proteobacterium HTCC2148	GPB2148_3061	665 - 1053
E4TSV1	Amidohydrolase	*Marivirga tractuosa* H-43[T]	Ftrac_2916	728 - 1112
E2LV52	Putative uncharacterized protein (Fragment)	*Moniliophthora perniciosa* FA553 / isolate CP02	MPER_11058	21 - 406
H1YB43	Amidohydrolase	*Mucilaginibacter paludis* TPT56[T]	Mucpa_6516	645 - 1032
H1YEC8	Amidohydrolase	*Mucilaginibacter paludis* TPT56[T]	Mucpa_2051	662 - 1051
F8CFH6	WD-40-like repeat-containing amidohydrolase	*Myxococcus fulvus* HW-1	LILAB_08375	703 - 1092
J1S5I4	TolB protein protein	*Myxococcus* sp. (contaminant ex DSM 436)	A176_3255	704 - 1092
Q1DG65	WD40-like repeat/amidohydrolase domain protein	*Myxococcus xanthus* DK 1622	MXAN_0075	626 - 1015
G6EA56	Amidohydrolase family protein	*Novosphingobium pentaromativorans* US6-1[T]	NSU_1227	647 - 1032
J2ZSX7	Periplasmic component of the Tol biopolymer transport system	*Novosphingobium* sp. AP12	PMI02_04678	653 - 1036
J3AHA5	Amidohydrolase, imidazolonepropionase	*Novosphingobium* sp. AP12	PMI02_01669	670 - 1053
F6IIH0	Amidohydrolase family protein	*Novosphingobium* sp. PP1Y	PP1Y_AT6612	646 - 1031
C6XW30	Amidohydrolase	*Pedobacter heparinus* HIM 762-3[T]	Phep_1901	653 - 1038

C6XVE6	Amidohydrolase	*Pedobacter heparinus* HIM 762-3^T	Phep_1803	645 - 1032
F0S567	Amidohydrolase	*Pedobacter saltans* Steyn 113^T	Pedsa_0402	642 - 1029
A6E852	WD40-like repeat/amidohydrolase domain protein	*Pedobacter* sp. BAL39	PBAL39_01167	647 - 1034
B4R7X0	Putative uncharacterized protein	*Phenylobacterium zucineum* HLK1^T	PHZ_c1089	696 - 1086
A6GAW3	WD40-like repeat/amidohydrolase domain protein	*Plesiocystis pacifica* SIR-1^T	PPSIR1_13080	685 - 1072
A4C1B1	Putative uncharacterized protein	*Polaribacter irgensii* 23-P^T	PI23P_11292	719 - 1102
A2U1R6	WD40-like repeat/amidohydrolase domain protein	*Polaribacter* sp. MED152	MED152_02795	711 - 1094
J0V4U4	Amidohydrolase	*Pontibacter* sp. BAB1700	O71_17026	540 - 927
B8PBL5	Predicted protein	*Postia placenta* Madison 698-R	POSPLDRAFT_88651	776 - 1173
Q15YP2	Amidohydrolase	*Pseudoalteromonas atlantica* T6c	Patl_0466	675 - 1067
JN228253	ι-carrageenan sulfatase	*Pseudoalteromonas carrageenovora* Psc^T	ι-CgsA	628 – 1016
Q3IKL4	Putative uncharacterized protein	*Pseudoalteromonas haloplanktis* TAC 125	PSHAa1171	650 - 1038
F3BMP2	Putative uncharacterized protein	*Pseudoalteromonas haloplanktis* ANT/505	PH505_bx00100	650 - 1038
E6RM22	Putative uncharacterized protein	*Pseudoalteromonas* sp. SM9913	PSM_A1853	650 - 1038
G7EW80	Putative uncharacterized protein	*Pseudoalteromonas* sp. BSi20311	P20311_2958	650 - 1038
G7F4P6	Putative uncharacterized protein	*Pseudoalteromonas* sp. BSi20429	P20429_2243	650 - 1038
G7FEZ5	Putative uncharacterized protein	*Pseudoalteromonas* sp. BSi20439	P20439_1796	650 - 1038
G7FMI0	Putative uncharacterized protein	*Pseudoalteromonas* sp. BSi20480	P20480_0813	643 - 1031
G7G6Q9	Putative uncharacterized protein	*Pseudoalteromonas* sp. BSi20495	P20495_3612	650 - 1038
G7EH21	Putative uncharacterized protein	*Pseudoalteromonas* sp. BSi20652	P20652_2058	650 - 1038
A4CBN2	Putative uncharacterized protein	*Pseudoalteromonas tunicata* D2^T	PTD2_18145	653 - 1041
I1DU95	Uncharacterized protein	*Rheinheimera nanhaiensis* E407-8^T	RNAN_0592	646 - 1034
F7NVZ8	Amidohydrolase, imidazolonepropionase	*Rheinheimera* sp. A13L	Rhein_1956	646 - 1034
I4WFD4	Amidohydrolase	*Rhodanobacter thiooxydans* LCS2^T	UUA_12540	656 - 1046
A4CMQ7	Putative uncharacterized protein	*Robiginitalea biformata* DSM 15991^T	RB2501_11502	715 - 1099
F8EFI8	Amidohydrolase	*Runella slithyformis* LSU 4^T	Runsl_1910	650 - 1041
D8QHN5	Putative uncharacterized protein	*Schizophyllum commune* H4-8	SCHCODRAFT_237900	840 - 1238
D8QHN9	Putative uncharacterized protein	*Schizophyllum commune* H4-8	SCHCODRAFT_70707	842 - 1238
F8Q6M6	Putative uncharacterized protein	*Serpula lacrymans lacrymans* S7.3	SERLA73DRAFT_94359	821 - 1217
F8P5B6	Putative uncharacterized protein	*Serpula lacrymans lacrymans* S7.9	SERLADRAFT_357566	821 - 1217
A1S1I4	Conserved hypothetical amidohydrolase	*Shewanella amazonensis* SB2B^T	Sama_0028	651 - 1039
A3CYI9	Amidohydrolase	*Shewanella baltica* OS155	Sbal_0015	188 - 575
G0APP2	Amidohydrolase	*Shewanella baltica* BA175	Sbal175_0015	652 - 1039
H1YVH9	Amidohydrolase	*Shewanella baltica* OS183	Sbal183_4304	652 - 1039
A6WH95	Amidohydrolase	*Shewanella baltica* OS185	Shew185_0011	652 - 1039
A9KU86	Amidohydrolase	*Shewanella baltica* OS195	Sbal195_0015	652 - 1039
B8E3Q8	Amidohydrolase	*Shewanella baltica* OS223	Sbal223_0015	652 - 1039
G6DWR2	Amidohydrolase	*Shewanella baltica* OS625	Sbal625DRAFT_1067	652 - 1039
E6SXW3	Amidohydrolase	*Shewanella baltica* OS678	Sbal678_0015	652 - 1039
A9EK13	Hypothetical amidohydrolase	*Shewanella benthica* KT99	KT99_03387	645 - 1032
Q12TB8	Amidohydrolase	*Shewanella denitrificans* OS217^T	Sden_0011	652 - 1039
D4ZD93	Amidohydrolase family protein	*Shewanella violacea* DSS12^T	SVI_0044	666 - 1053
Q08A43	Amidohydrolase	*Shewanella frigidimarina* NCIMB 400	Sfri_0009	650 - 1037
B0TLB3	Amidohydrolase	*Shewanella halifaxensis* HAW-EB4^T	Shal_0010	650 - 1038
A3Q8T8	Amidohydrolase	*Shewanella loihica* PV-4^T	Shew_0013	658 - 1045
Q8EKS3	Putative uncharacterized protein	*Shewanella oneidensis* MR-1^T	SO_0017	639 - 1026
A8GYF2	Amidohydrolase	*Shewanella pealeana* ANG-SQ1^T	Spea_0010	658 - 1040
B8CH79	Amidohydrolase:Amidohydrolase-like protein	*Shewanella piezotolerans* WP3^T	swp_0023	650 – 1038
E6XNJ7	Amidohydrolase	*Shewanella putrefaciens* 200	Sput200_0009	652 - 1039
A4Y1B2	Amidohydrolase	*Shewanella putrefaciens* CN-32	Sputcn32_0009	652 - 1039
A8FP58	Amidohydrolase	*Shewanella sediminis* HAW-EB3^T	Ssed_0018	664 - 1051
A0KR45	Amidohydrolase	*Shewanella* sp. ANA-3	Shewana3_0019	652 - 1039
Q0HPC4	Amidohydrolase	*Shewanella* sp. MR-4	Shewmr4_0011	652 - 1039
Q0I0T8	Amidohydrolase	*Shewanella* sp. MR-7	Shewmr7_0011	652 - 1039
A1RDW0	Amidohydrolase	*Shewanella* sp. W3-18-1	Sputw3181_0009	652 - 1039
F7RUR4	Amidohydrolase	*Shewanella* sp. HN-41	SOHN41_04102	652 - 1039
B1KCZ0	Amidohydrolase	*Shewanella woodyi* MS32^T	Swoo_0015	660 - 1047
G2II00	Putative amidohydrolase	*Sphingobium* sp. SYK-6	SLG_22480	661 - 1051
Q1GU57	Amidohydrolase	*Sphingopyxis alaskensis* RB2256^T	Sala_1098	663 - 1053
E3FKF4	Amidohydrolase	*Stigmatella aurantiaca* DW4/3-1	STAUR_0118	666 - 1054
Q6SFD8	Amidohydrolase domain protein	Uncultured marine bacterium 581	MBMO_EBAC000-69B03.30	662 - 1054
Q8RTU1	Putative uncharacterized protein EBAC000-65D09.41	Uncultured proteobacterium	EBAC000-65D09.41	662 - 1054
A6EP70	WD40-like repeat/amidohydrolase domain protein	Unidentified eubacterium SCB49	SCB49_13845	655 - 1044
F0C126	Amidohydrolase, imidazolonepropionase	*Xanthomonas gardneri* Strain 101^T	XGA_0530	702 - 1084

The accession numbers derived from the UniProt data bank except for the ι-carrageenan sulfatase which is from GenBank. The limits of the amidohydrolase module are those used in the phylogenetic analysis. The superscript letter T indicates the strain's type.
